# Prediction of cooperative homeodomain DNA binding sites from high-throughput-SELEX data

**DOI:** 10.1093/nar/gkad318

**Published:** 2023-04-28

**Authors:** Brittany Cain, Jordan Webb, Zhenyu Yuan, David Cheung, Hee-Woong Lim, Rhett A Kovall, Matthew T Weirauch, Brian Gebelein

**Affiliations:** Department of Biomedical Engineering, University of Cincinnati, Cincinnati, OH 45221, USA; Division of Developmental Biology, Cincinnati Children's Hospital Medical Center, 3333 Burnet Ave, MLC 7007, Cincinnati, OH 45229, USA; Department of Molecular Genetics, Biochemistry and Microbiology, University of Cincinnati College of Medicine, Cincinnati, OH 45267, USA; Department of Molecular Genetics, Biochemistry and Microbiology, University of Cincinnati College of Medicine, Cincinnati, OH 45267, USA; Graduate Program in Molecular and Developmental Biology, Ci ncinnati Children's Hospital Research Foundation, Cincinnati, OH 45229, USA; Division of Biomedical Informatics, Cincinnati Children's Hospital Medical Center, Cincinnati, OH 45229, USA; Department of Pediatrics, University of Cincinnati College of Medicine, Cincinnati, OH 45229, USA; Department of Molecular Genetics, Biochemistry and Microbiology, University of Cincinnati College of Medicine, Cincinnati, OH 45267, USA; Department of Pediatrics, University of Cincinnati College of Medicine, Cincinnati, OH 45229, USA; Divisions of Human Genetics, Biomedical Informatics and Developmental Biology, Center for Autoimmune Genomics and Etiology (CAGE), Cincinnati Children's Hospital Medical Center, Cincinnati, OH 45229, USA; Division of Developmental Biology, Cincinnati Children's Hospital Medical Center, 3333 Burnet Ave, MLC 7007, Cincinnati, OH 45229, USA; Department of Pediatrics, University of Cincinnati College of Medicine, Cincinnati, OH 45229, USA

## Abstract

Homeodomain proteins constitute one of the largest families of metazoan transcription factors. Genetic studies have demonstrated that homeodomain proteins regulate many developmental processes. Yet, biochemical data reveal that most bind highly similar DNA sequences. Defining how homeodomain proteins achieve DNA binding specificity has therefore been a long-standing goal. Here, we developed a novel computational approach to predict cooperative dimeric binding of homeodomain proteins using High-Throughput (HT) SELEX data. Importantly, we found that 15 of 88 homeodomain factors form cooperative homodimer complexes on DNA sites with precise spacing requirements. Approximately one third of the paired-like homeodomain proteins cooperatively bind palindromic sequences spaced 3 bp apart, whereas other homeodomain proteins cooperatively bind sites with distinct orientation and spacing requirements. Combining structural models of a paired-like factor with our cooperativity predictions identified key amino acid differences that help differentiate between cooperative and non-cooperative factors. Finally, we confirmed predicted cooperative dimer sites *in vivo* using available genomic data for a subset of factors. These findings demonstrate how HT-SELEX data can be computationally mined to predict cooperativity. In addition, the binding site spacing requirements of select homeodomain proteins provide a mechanism by which seemingly similar AT-rich DNA sequences can preferentially recruit specific homeodomain factors.

## Graphical Abstract

**Graphical Abstract:**
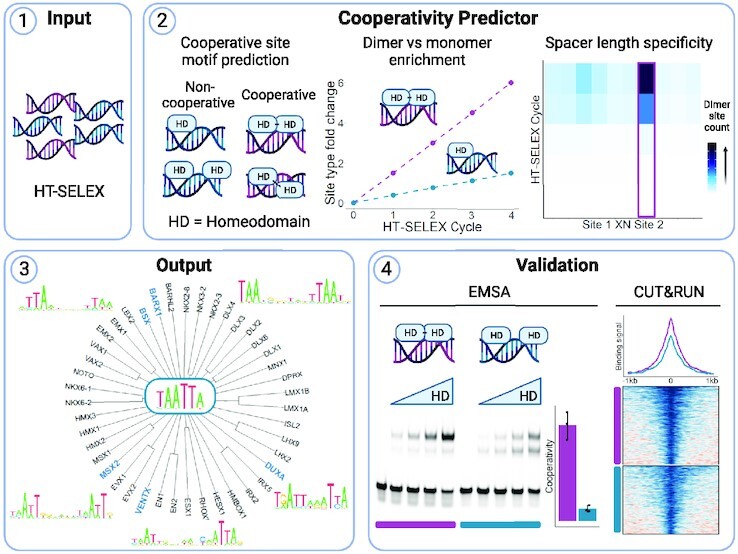
Cooperativity Predictor utilizes HT-SELEX data to predict if a homeodomain (HD) transcription factor has the capability to bind to DNA cooperatively and generates cooperative dimer site DNA motifs for each prediction. The pipeline was validated through quantitative EMSAs and analysis of available genomic binding data.

## INTRODUCTION

The differential control of gene expression is fundamental for the specification of distinct cell types during development. At the transcriptional level, sequence-specific transcription factors (TFs) regulate gene expression by binding *cis*-regulatory modules (CRMs) to inhibit or promote RNA polymerase activity through the recruitment of co-factors. Hence, the binding of TFs to their correct regulatory regions is vital for proper development. Many CRMs are conserved across metazoans by sequence and/or function (1,2), and not surprisingly, mutations in these regulatory regions have been associated with developmental, autoimmune, and cardiovascular diseases as well as cancer (3). Thus, it is essential to define the DNA binding characteristics of TFs to better understand how each TF accurately regulates target gene expression and ultimately cellular fates.

Metazoan genomes encode numerous sequence-specific TFs that participate in gene regulation (4). Many of these TFs can be categorized into families based on conserved DNA binding domains, and often members within a family bind highly similar DNA sequences. The homeodomain (HD) family is one of the largest TF families, consisting of almost 200 family members in humans (5). HD proteins have been separated into distinct classes based on conserved sequence features such as the presence of additional DNA binding domains in the Paired, Pou, and CUT classes; amino acid insertions within the HD in the three amino acid loop extension (TALE) and Prospero classes; and conserved amino acid motifs in the NK-like, HOX-like, and Paired-like classes (6). All members of the HD family encode a helix-turn-helix DNA binding domain that contains three alpha helices. Structural and mutation studies revealed that HD proteins use this DNA binding domain to mediate direct contact to a AT-rich core DNA motif such as TAATNN through largely conserved residues. Within the typical 60 amino acid HD, the conserved Arginine 5 (R5) and R3 residues in the N-terminal Arginine-Rich Motif (ARM) of the first alpha-helix contact the first and second DNA positions on the minor groove of DNA, and the common N51 and I/V47 in the third alpha-helix contact the third and fourth DNA positions in the major groove of DNA (7,8). In addition, the 50th residue of the HD, which can vary between different residues including Q50, K50 and S50, contacts the fifth and sixth DNA positions on the major groove of DNA, and thereby contributes to the binding site specificity of these DNA positions (6–10). Additional amino acids that vary between HDs can impact DNA binding specificity (10), but the above listed residues are thought to be the dominant drivers of site specificity. Given the high-degree of sequence conservation in residues that contact DNA between HD factors, it is not surprising that the majority of HD TFs bind similar AT-rich sequences *in vitro* (6,11). However, in sharp contrast to their similar *in vitro* DNA binding activities, most HD TFs regulate distinct developmental pathways *in vivo*, and the mechanisms by which members of the HD family differentiate between like DNA sequences to bind and regulate distinct targets is not well understood (12,13).

One solution to the specificity problem is that TFs can form homo- and heterodimer complexes that bind distinct sequences and/or lengthen the recognition site. For example, while the Hox TFs that specify anterior–posterior identities in metazoans have relatively low sequence specificity as monomers, Hox TFs form heterodimer complexes on DNA with the PBX factors, and thereby increase their DNA binding specificity (14). Further, some Paired-like HD factors, which contain a HD most homologous to those found in PAX factors but lack the accompanying paired domain (6), bind as homodimers and heterodimers with other members of the Paired-like class to increase specificity (15) and alter transcriptional output (9,16). Likewise, TFs outside of the HDs including the bZIP (17), bHLH (18) and nuclear receptor (19) families bind DNA as obligate homodimers and/or heterodimers, and thereby lengthen the DNA recognition sequence. Many of these dimeric TF complexes are considered cooperative since the binding of the second protein is largely facilitated or dependent upon the binding of the first protein.

Given the central importance of TFs in regulating gene expression, a great deal of effort has been put toward defining the DNA sequence binding preferences of each TF. *In vivo* methods such as chromatin immunoprecipitation sequencing (ChIP-seq) and cleavage under targets and release under nuclease (CUT&RUN) can identify genomic regions bound by a target TF. However, both methods require either a high-quality antibody for each TF or expression of a tagged version of the protein which may alter TF activity. There are also an endless number of combinations of biological tissues, developmental stages, and TFs to test with these methods. Further, the most highly enriched motif in a ChIP-seq experiment is not always the motif for the TF examined in the assay. An alternative approach has been to use *in vitro* assays such as protein binding microarrays (PBMs) and high throughput sequencing of systematic evolution of ligands by exponential enrichment (HT-SELEX) assays, which have been used to define the sequence preferences for hundreds of transcription factors in a standardized synthetic environment (11,20–23). A strength of PBMs is that microarrays can be designed to contain each 8mer sequence 32 times and the assay provides semi-quantitative binding information since binding is measured using a fluorescent protein that does not require amplification. However, PBMs have less utility in systematically identifying motifs longer than 8–10 bps. In contrast, HT-SELEX uses random sequences that are between 20 and 40 bps and can detect the binding of longer motifs, including those bound by TF multimers. Until recently, SELEX assays only obtained qualitative binding information unlike PBMs. However, Rube *et al.* developed the machine learning algorithm, ProBound, to compute quantitative TF binding models and absolute affinities from a modified SELEX-seq protocol, kD-seq (24).

As expected, analysis of the TF binding motifs for human and mouse HD factors using the PBM and HT-SELEX methods revealed highly similar AT-rich monomer binding sites for most proteins (11,20). In addition, the HT-SELEX assays showed that some HD TFs enriched for both monomer and dimer sites, suggesting that a subset of HD TFs may gain DNA binding specificity by binding DNA as homodimers (11). Moreover, as part of a previous study on how the mouse HD TF, Gsx2, regulates gene expression during forebrain development, we re-analyzed the human GSX2 HT-SELEX data that was originally found to only enrich for a monomer site, and identified a significantly enriched dimer site consisting of 2-TAAT motifs spaced 7 bp apart (25). We subsequently used electrophoretic mobility shift assays (EMSAs) to show that Gsx2 cooperatively bound this novel dimer site. This capability of Gsx2 to regulate gene expression through both short monomer sites and longer dimer sites with specific spacing and orientation requirements enhances the DNA binding specificity of this factor compared to other HD factors. In this study, we broadly assess the prevalence of homodimer cooperativity in the HD family by first developing a computational approach to predict cooperative HD DNA binding from existing HT-SELEX data, and second, by using quantitative EMSAs to systematically test a subset of the HDs for cooperative DNA binding. Importantly, our findings reveal how the relative rate of binding site enrichment within HT-SELEX assays and the selection for a specific spacer length within the dimer site can be utilized to accurately predict cooperative TF binding. Further, we show that a subset of the Paired-like class of HDs cooperatively binds palindromic dimer sites spaced 3 bp apart, and we explore how amino acid differences between paired-like HDs impact cooperative DNA binding. In contrast to the consistent spacing requirements of the paired-like class, we found that a subset of other HD proteins bind cooperatively to unique binding site arrangements. We then re-analyzed available genomic data and found evidence that these dimeric sites are being bound *in vivo* for many of these HDs. Taken together, these findings highlight how HT-SELEX data can be analyzed to identify members of the HD family that enhance their DNA binding specificity by forming cooperating complexes on distinct dimer DNA binding site configurations.

## MATERIALS AND METHODS

### HT-SELEX TF binding dataset acquisition and analysis

**Figure 1.**
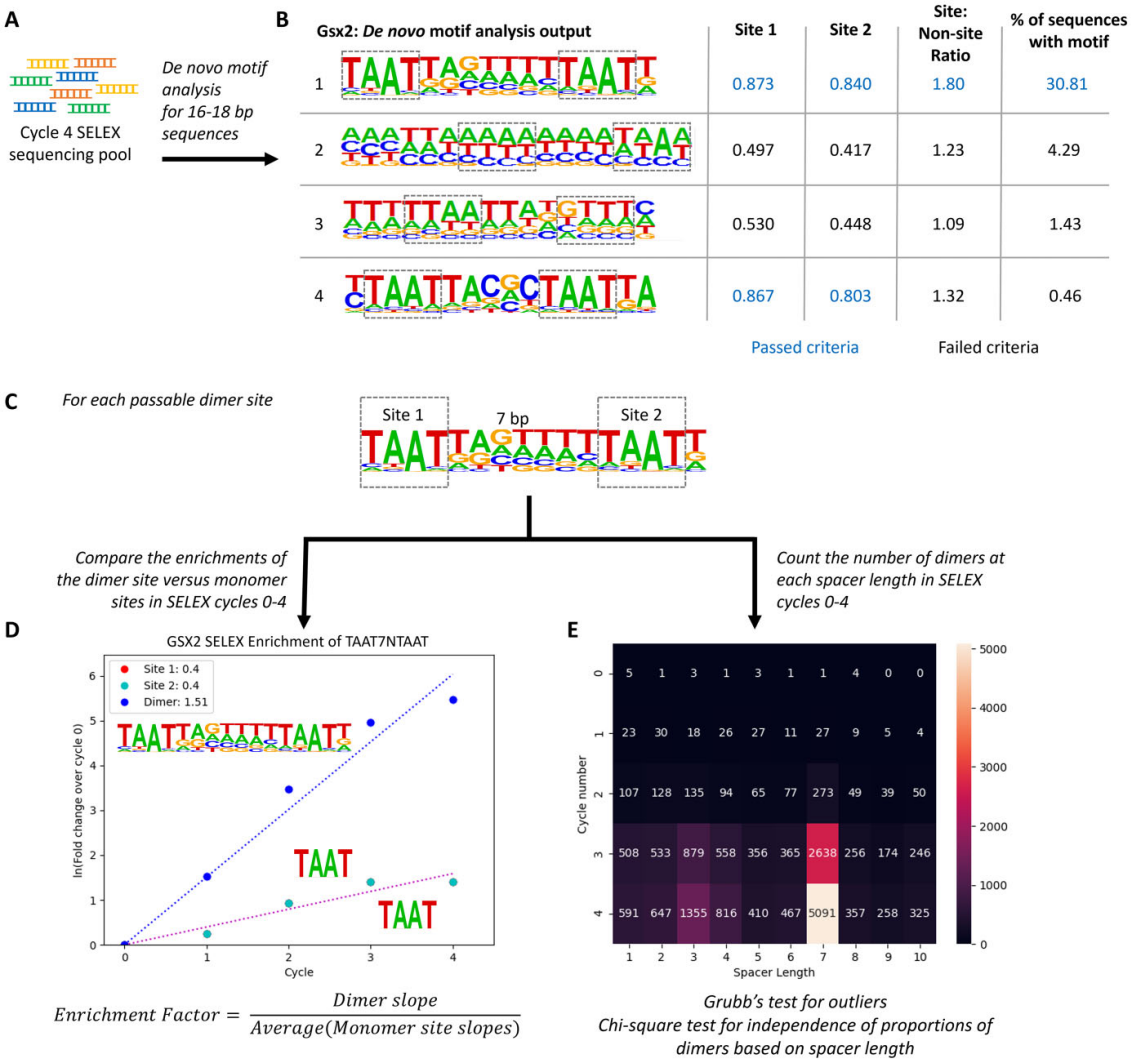
The Cooperativity Predictor uses dimer to monomer site enrichment rates and spacer length constraints between sites to predict the cooperativity of HD TFs from HT-SELEX data. (**A**) A *de novo* motif analysis for long motifs (16–18bps) was performed using the cycle 4 sequencing pool of HT-SELEX for each HD TF. (**B**) The two 4mers with the highest information content within each PWM were selected to define each site. Each generated PWM was then interrogated for dimer sites using the following criteria: First, the information content of the two 4mer sites (Site 1 and Site 2) had to be greater than 0.6. Second, the information content of the 4mer sites must be 1.5 times greater than those of the surrounding regions. Third, the motif had to be present in at least 5% of the sequences in the cycle 4 sequencing pool. Note, those numbers shown in bold blue text passed the selected criterium. (**C**) For each dimer site that passed the selection criteria, the two 4mers and the spacer length were defined. (**D**) The percentage of sequences with dimer sites and monomer sites were determined after each cycle and used to calculate the fold changes of the dimer and monomer sites. Given the exponential nature of cycle amplification in PCR, the data was linearized through a natural log transformation. The enrichment factor is defined as the slope of the dimer enrichment over the average slopes of the 2 individual 4mer sites. (**E**) COSMO was used to count the number of dimers composed of the 2 4mers at each spacer length. Note, cycle 0 is the initial library prior to any selection process. The specific spacer length enrichment in cycle 4 was tested via a Grubb's test for outliers, and the dimer proportions between the initial library and cycle 4 were compared with a chi-square test for independence.

The *in vitro* HT-SELEX data analyzed using the Cooperativity Predictor was acquired from European Nucleotide Archive under accession entry, PRJEB3289 (11). The run accession IDs and download links for all the HD TFs analyzed from the HT-SELEX study are listed in Supplementary Table S1. All SELEX datasets performed with a HD DNA binding domain that had an available initial library and utilized a ligand 20 bp or longer were analyzed with the Cooperativity Predictor pipeline (code available at https://github.com/cainbn97/Cooperativity_predictor). The steps and rationale for the HD analysis criteria within the Cooperativity Predictor pipeline are provided in the Results and Figure 1. In brief, the general steps were as follows: (i) Homer *de novo* motif analysis for 16–18 bp motifs was performed using 50 000 randomly selected sequences from the fourth round of HT-SELEX selection to define potential dimer sites. (ii) The two highest information content 4mer sequences that were at least 2 bp apart were defined as core 4mer motifs. The 4mer PWM was then generated with the seq2profile.pl script in Homer. (iii) The rate and ratio of dimer to monomer site enrichment after each cycle were calculated using the initial library and the data from each HT-SELEX cycle. (iv) COSMO determined the number of dimers that were present at each spacer length after each HT-SELEX cycle (26). (v) The enrichment of a single spacer length from the fourth cycle HT-SELEX selection was tested with a Grubb's test for outliers and the change in dimer proportion between the initial library and the fourth cycle of SELEX selection was tested with a chi-square test for independence. The pipeline results for the HD family are provided in Supplementary Figure S3. PWMs of the enriched long motifs that contained dimer sites are listed in Supplementary Table S2. The Jaspar formatted PWMs (jpwm) used in the COSMO analysis can be found in Supplementary Table S3. The multiple sequence alignments were generated using the HD amino acid sequences used in the HT-SELEX assay with the MSA package (27) and the phylograms were plotted with the ape package (28) in R.

### Genomic TF binding dataset acquisition and analysis

For the *in vivo* genomic data analysis, each respective ChIP-seq and CUT&RUN dataset was acquired from Gene Expression Omnibus using the accession IDs listed in Supplementary Table S4. For consistency, all genomic binding assay datasets were reprocessed from their raw fastq files. The sequencing data underwent adaptor trimming with Cutadapt (29) and quality control with FastQC via the wrapper, TrimGalore. Results were mapped to hg19 or mm10 using Bowtie2 (30), and duplicates were removed with Picard (Broad Institute). All reads longer than 150bp were removed prior to further analysis in CUT&RUN processing. Peaks were called with MACS3 using reads across all replicates (31). Peaks were extended to 1kb on each side of the MACS3 summits with BEDTools (32), and motif densities across these regions were determined via Homer (33). Datasets in which less than 1200 peaks were called had a moving average smoothing function applied to the motif densities to increase readability of overall trends. The log2ratios of the binding signals between the IP experiment and the IgG control were calculated and plotted using deepTools (34). The number of dimer sites at each spacer length within the called narrow peaks from MACS3 (not the extended peak summits) was determined via COSMO (26).

### Molecular cloning

TF sub-fragments containing HD and flanking sequences were PCR amplified from cDNA clones obtained from Genscript. Oligonucleotide sequences used to amplify the TF sub-fragments are listed in Supplementary Table S5 (IDT). Accuzyme DNA polymerase (Bioline) or GoTaq Master mix (Promega) was used to PCR amplify regions. The Isx sub-fragment was synthesized through Genscript rather than PCR amplified from its cDNA clone. The amino acid sequences of the TF sub-fragments are listed in Supplementary Table S6. TF sub-fragments were ligated into a bacterial expression vector with T4 DNA ligase (NEB). The bacterial expression vector was either pET-14b (Novagene), which contains an N-terminal 6xHis-tag, or a modified version of pET-14b called pET-14P, in which additional restriction enzyme sites and a PreScission Protease site was inserted between the His-tag and TF coding sequence in place of the original thrombin cleavage site. The vector used for each TF is indicated in Supplementary Table S6 and the full sequence of pET-14P is provided in the supplementary information. Isx, VENTX, Gsx1, MSX1, Msx2 and HESX1 were cloned in between BamHI and NotI sites. Cart1, Alx4, Arx, Barx1, Bsx and Gsx2 were cloned in between NdeI and XhoI sites. All constructs were confirmed via DNA sequencing.

### Protein purification and electrophoretic mobility shift assays (EMSA)

TF sub-fragments containing HD and flanking regions were purified either from BL21 (DE3) *E. coli* under native conditions without dialysis using Ni-chromatography as previously described (35) or via the following method: The expression vector was transformed into C41DE3 (Sigma-Aldrich) *E. coli* and bacteria were grown in autoinduction media at 37°C for 3 h and then cooled to 20°C overnight. The cultures were harvested by centrifugation. Cell pellets were resuspended in binding buffer (1XBB; 20 mM Tris pH 8, 500 mM NaCl, 5 mM Imidazole), lysed by sonication, cleared by centrifugation, and loaded onto Ni^2+^ beads. Beads were then loaded into a gravity column, washed with 1XBB with 0.1% Triton twice and 1XBB with 0.1% NP40 once. Protein was eluted using 1XBB with 0.1% NP40 and 0.5 M Imidazole. The eluted protein was dialyzed, and the His-tag cleaved with PreScission Protease (GE Healthcare) per the manufacturer's protocol. Protein was further purified via cation exchange and size exclusion chromatography. Finally, the protein was concentrated in a buffer containing 20 mM MES pH 6, 150 mM NaCl, 1% ethylene glycol and 0.1 mM TCEP.

TF purity was confirmed by SDS-PAGE with GelCode blue staining (Thermo Scientific) (Supplementary Figure S11). Protein concentrations were determined by Bradford assays (Bio-rad). EMSA probes were prepared as previously described (36), and the sequences used for EMSA probes are listed in Supplementary Table S7. EMSA binding reactions were prepared as described previously (35) and incubated at room temperature for 20 min before being run on a 4% polyacrylamide gel for 2 hours at 150V. Gels were imaged via Li-Cor Odyssey CLx scanner and monomer, dimer, and free probe bands were quantified via the Li-Cor image studio software. Calculation of cooperativity via the Tau factor was performed as previously described (15). In brief, the Tau factor calculation is based on the dissociation constants derived from the equilibrium reactions of a single protein binding to DNA (*K*_d1_) and the binding of second protein to the protein-DNA complex (*K*_d2_).


$$\begin{equation*}\tau \ = \ \frac{{4\ \left[ {{P}_2D} \right]\left[ D \right]}}{{{{\left[ {PD} \right]}}^2}}\end{equation*}$$

In this equation, $[ {{P}_2D} ]$ represents the proportion of probe bound as a dimer, $[ {PD} ]$ indicates the proportion of probe bound as a monomer, and $[ D ]$ is the proportion of unbound probe. The way in which the binding of the first protein facilitates the binding of the second protein is the coefficient of *K*_d2_/*K*_d1_, or the Tau factor.

### Modeling HD variants in paired-like subclass

Variants were modeled on the *Drosophila* paired HD (PDB: 1FJL) (37) using the mutagenesis wizard in Pymol v2.2.0 (Schrödinger). The disks and colors indicate pairwise overlap of atomic van der Waals radii. Large red disks indicate significant van der Waals overlap, whereas green and yellow disks represent minor overlap. The rotamer that demonstrated the least amount of clashing was modeled for each of the variants.

## RESULTS

### Predicting transcription factor cooperativity using HT-SELEX data

Inspired by our prior successful demonstration of the cooperative binding of GSX2 to bioinformatically identified dimeric DNA sites and the large amount of available HT-SELEX data for HD TFs, we developed a computational pipeline, termed the Cooperativity Predictor, that examines the growth rate of dimer versus monomer motif enrichment and the preference for a specific spacer length between sites within each HT-SELEX cycle to predict cooperative binding behavior. First, we used Homer *de novo* motif analysis (33) to identify potential dimer sites by generating position weight matrices (PWMs) representing the most enriched long motifs (i.e. 16 or 18mers) after the fourth cycle of HT-SELEX compared to the initial unselected DNA library (Figure 1A). Our rationale is that enriched long motifs are likely to contain more than one HD site, consistent with a potential dimer binding site. Using GSX2 as an example, Homer identified four enriched long motifs to consider as potential dimer sites (Figure 1B). However, automating this unbiased approach revealed two fundamental challenges.

First, the HT-SELEX method does not directly differentiate between monomer versus dimer binding. Thus, enriched long motifs could either consist of a single TF (i.e. a monomer) bound to a single long site or represent two TFs (i.e. a dimer) bound to two independent sites. To help discriminate between long monomer sites versus dimer sites, we first identified the two non-overlapping 4mer sequences that have the highest information content within each motif (boxed in Figure 1B). 4mers were used in this analysis as structural studies have revealed that conserved residues in the HD primarily mediate direct contact to a core 4mer sequence (7,8). We then required the non-overlapping 4mers to be at least 2 bp apart and the average information content for each 4mer to be >0.6 (see Supplementary Figure S1A-B,E for how we established the 0.6 threshold). Importantly, we used all available human HD datasets to establish the information content thresholds (Supplementary Figure S1E). Applying the site information content threshold to the four enriched GSX2 motifs revealed that only the first and fourth motifs had sufficient information content within each 4mer sequence to pass this criterion (Figure 1B).

The second challenge is that HT-SELEX relies upon non-linear sequence amplification due to the polymerase chain reaction (PCR) between cycles. Thus, enriched dimer motifs may represent relatively rare binding events that have been artificially enriched due to PCR bias. In general, if a sequence is over-amplified, the replicate 20mers and 30mers produce motifs with high information content across the entire PWM and not just the core 4mer sequences (Supplementary Figure S1D). To eliminate such motifs, we required the information content within the 4mers to be 1.5 times greater than the surrounding flanking and spacer sequences (see Supplementary Figure S1C–E for how we empirically established 1.5 as a threshold). In essence, by requiring sufficient variability in information content between the nucleotide positions of the PWM, we ensure that the motif was generated by multiple distinct sequences rather than by a few over-amplified sequences. When applying this filter to the GSX2 motifs, we found that the fourth motif, which had sufficient information content within the 4mers to pass the first criterion, failed this criterion due to very little variation in information content between the 4mer sites and surrounding sequence. Consistent with this motif representing a relatively rare binding event, we found that <1% of the sequences after the fourth selection cycle contained this motif. In contrast, GSX2 motif 1, which encodes the previously identified cooperative GSX2 binding site (25), passed this second criterion and was found in over 30% of the selected sequences. To ensure the exclusion of rare binding events prior to further analysis, we only considered dimer sites that occurred in at least 5% of the cycle 4 HT-SELEX datasets (Figure 1B). A visual demonstration of how these thresholds impacted dimer site selection of a sampled set of TFs is shown in Supplementary Figure S2.

Having selected candidate dimer sites using motifs from the fourth HT-SELEX cycle, we next incorporated HT-SELEX’s multiple rounds of selection to calculate the rate of enrichment of dimer sites versus monomer binding sites. Our rationale is that if a TF forms a cooperative homodimer on DNA, the added protein–protein interactions in dimer binding and/or DNA conformation changes triggered by the binding of the first protein will increase complex stability (38,39). Thus, cooperative dimer sites should enrich at a faster rate through the HT-SELEX cycles than monomer sites. To apply this idea, we compared the enrichment rate of dimer versus monomer sites through each of the four HT-SELEX cycles. Since dimer sequences contain two binding sites that would also be considered as monomer sites, we masked sequences that Homer predicted contained a dimer site when calculating the rate of monomer site enrichment. Using this approach, we calculated the number of sequences after each selection cycle that do not have a dimer site but contain at least one of the selected 4mer sequences (e.g. TAAT for GSX2). We defined the fold change as the ratio of the number of sites after each SELEX cycle versus the initial library. As sites undergo exponential enrichment due to SELEX selection and PCR amplification (23), we linearized the fold enrichment by taking the natural logarithm. Enrichment slopes of dimer and monomer sites were calculated, and the enrichment factor was defined as the ratio of the dimer site enrichment slope and the average of the two monomer site enrichment slopes. In this analysis, a positive prediction of cooperativity was defined by an enrichment factor >2 as the probability of finding the dimer site was more likely than finding the two monomer sites. Applying this analysis to the GSX2 HT-SELEX data using the predicted dimer motif in Figure 1C and the 4mer sites (TAAT) revealed a calculated slope of 1.51 for the dimer site versus a slope of 0.4 for the 4mer sites (Figure 1D). Hence, the enrichment factor for GSX2 was calculated to be 3.78. Thus, the rate of GSX2 dimer to individual site enrichment across the HT-SELEX cycles positively correlates with GSX2’s ability to cooperatively bind dimer sites.

An alternative explanation for the fast rate of dimer site enrichment is that HT-SELEX may simply select sequences with any two sites at a faster rate than those sequences with only a single site. Since cooperative TFs typically bind dimer sites with a specific spacer length (5,11,38), we next assessed for the selective enrichment of specific spacer lengths between sites using combinatorics of stereospecific motif orientation (COSMO) (26). COSMO systematically counts the number of sequences with two sites at variable spacer lengths. We applied this program to count the number of dimers consisting of the 4mers at each spacer length after each round of HT-SELEX (Figure 1E). The preference of a specific spacer length in the cycle 4 sequencing pool was tested with a Grubb's test for outliers, and the proportions of dimers at each spacer length were compared between the initial library and cycle 4 using a chi-square test for independence. If *P* < 0.05 for both tests, the TF was considered to select for a dimer site with a specific spacer length which positively correlates with cooperativity. Applying COSMO to the GSX2 HT-SELEX data revealed that only TAAT sites separated by a 7 bp spacer length passed the Grubb's outlier and chi-square test for independence (Figure 1E). In summary, the Cooperativity Predictor pipeline considers both the added stability that cooperativity provides using the rate of site enrichment as well as spacer length specificity using COSMO to predict cooperativity using HT-SELEX data.

### Cooperativity predictor identified previously known and novel cooperative DNA binding sites

**Figure 2.**
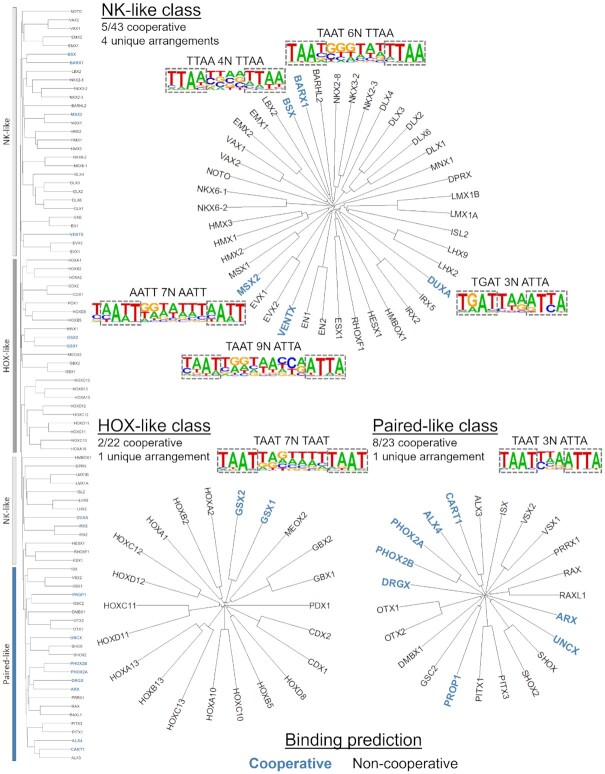
Cooperativity predictions across HD subclasses. (Left side) Protein sequence divergence among the 88 human HDs was used to separate the family into the NK-like, HOX-like, and Paired-like subclasses as defined by Bürglin and Affolter (6). Prior studies had not revealed that NK-like and HOX-like family members bind DNA as cooperative homodimers, except for Gsx2 (members categorized by gray dividers). In contrast, some members of the Paired-like family (blue divider) were previously shown to cooperatively bind DNA (15). The Cooperativity Predictor identified TFs that are predicted to bind cooperatively (bolded and colored in blue text). (Top) Five members of the NK-like class were predicted to bind cooperatively to four unique binding arrangements. BARX1 and MSX2 were predicted to bind cooperatively to similar DNA binding sequences. (Bottom left) Two of the 22 members of the HOX-like class were found to bind to 1 unique binding arrangement, in which close homologues GSX1 and GSX2 both were predicted to bind cooperatively to a similar site. (Bottom right) Eight of the 23 Paired-like factors were predicted to bind palindromic sites 3 bp apart.

The HD family has been separated into distinct classes based on defining features that include the presence of additional DNA binding domains (i.e. the Paired, Pou, and CUT domains) (6). As these TFs are well known to use both DNA binding domains to bind dimer sites, we focused our analysis on HD TFs that lack well characterized domains that contribute to dimer binding. Mining the available human HT-SELEX data, we applied the Cooperativity Predictor to 88 human HD proteins (Figure 2; Supplementary Figure S3). Of the 88 HD proteins analyzed, 28 (32%) had at least one detectable dimer site within the long motif (16–18 bp) identified by Homer after cycle 4 (Supplementary Figure S3), and of these, 15 had specific spacer requirements within the highly enriched dimer site (Figure 2). In addition, 24 mouse HD proteins have HT-SELEX datasets that met our analysis requirements. Of these, 16 were orthologues to the above human HD factors. Analysis of the mouse data revealed that 7 of the 24 mouse TFs were predicted to bind cooperatively (Supplementary Figure S3). Importantly, we found that the cooperativity predictions of the mouse and human orthologs were consistent for 14 of the 16 orthologues tested. For example, like the human CART1 and ALX4 TFs, the mouse orthologs Alx1 and Alx4 enriched for a sequence containing two palindromic sites spaced 3bp apart, which is consistent with past studies (9,40,41) (Supplementary Figure S3). In addition, the mouse and human orthologs of ARX and UNCX were predicted to bind cooperatively to highly similar sites, and there were 10 cases in which both orthologs were not predicted to bind DNA cooperatively (Supplementary Figure S3).

To define the relationships between these HD TFs, we used the human HD factors to generate phylogenetic trees based on multiple sequence alignments of the protein regions tested in the HT-SELEX assays and classified these proteins into the NK-like, HOX-like, and Paired-like subclasses as described by Burglin and Affolter (6) (Figure 2). There were select human TFs in each subclass that were predicted to bind DNA cooperatively (Figure 2). Notably, in some cases, the PWMs were largely identical, although differences in information content caused the selected top 4mers to shift by one or two nucleotide positions. For example, the BARX1 and MSX2 NK-like factors bound nearly identical PWMs, but the consensus sequences defined by the highest information content were TAAT6NTTAA and AATT7NAATT respectively (Supplementary Figure S4A). Thus, these motifs were considered one unique binding arrangement. In total, five members of the human NK-like class were predicted to bind cooperatively to four unique binding arrangements with spacer lengths between 3 and 9 bp (Figure 2, top). Most of these NK-like TFs are not near one another in the phylogram tree and the unique binding arrangements found suggest that they independently obtained their dimer binding site preferences.

The classic HOX factors that specify segment identities along the anterior-posterior axis are known to bind in complex with PBX and MEIS factors to increase TF-DNA specificity by lengthening the recognition sequences (14,42,43). However, members of the HOX-like subclass are not thought to bind as cooperative homodimers. Consistent with this idea, the Cooperativity Predictor did not predict any cooperative dimer sites for the human HOX-like factors apart from GSX1 and GSX2 (Figure 2, bottom left). Moreover, we previously confirmed that the mouse Gsx2 protein cooperatively bound this predicted dimer sequence biochemically *in vitro* and via CUT&RUN analysis *in vivo*, and we found that the fly GSX homologue, Ind, cooperatively binds this dimer motif (25). Hence, the prediction that the close homologue GSX1 also cooperatively binds to a nearly identical enriched homodimer site suggests this activity is a conserved feature of the GSX/Ind HD TF family.

Several members of the Paired-like class of HD factors have been previously shown to cooperatively bind palindromic DNA sequences spaced 3 bp apart (15). Here, we greatly expanded the number of these factors with this activity by predicting that 8 of the 23 human members of the Paired-like class cooperatively bind to highly similar palindromic 4mer sequences (Figure 2, bottom right; Supplementary Figure S4B). The NK-like, HOX-like, and Paired-like HD cooperativity predictions revealed a diverse set of spacing requirements from 3 bp for the Paired-like HDs to 4 bp for BSX to 9 bp for VENTX. Intriguingly, however, the unrelated BARX1 and MSX2 NK-like HDs and the GSX1 and GSX2 HOX-like HDs shared highly similar dimer sites (TAAT7NTAAT) (Supplementary Figure S4A). Taken together, these data suggest that a subset of HD TFs utilize dimer formation to increase DNA binding specificity through the binding of differentially spaced sites, yet others may still compete with one another for specific dimer sites under certain cellular contexts. However, to our knowledge, only three of these factors (CART1, ALX4, and GSX2) have been experimentally shown to biochemically bind such sequences in a cooperative manner.

### Experimental validation of spacer-dependent cooperativity of homeodomains in vitro

**Figure 3.**
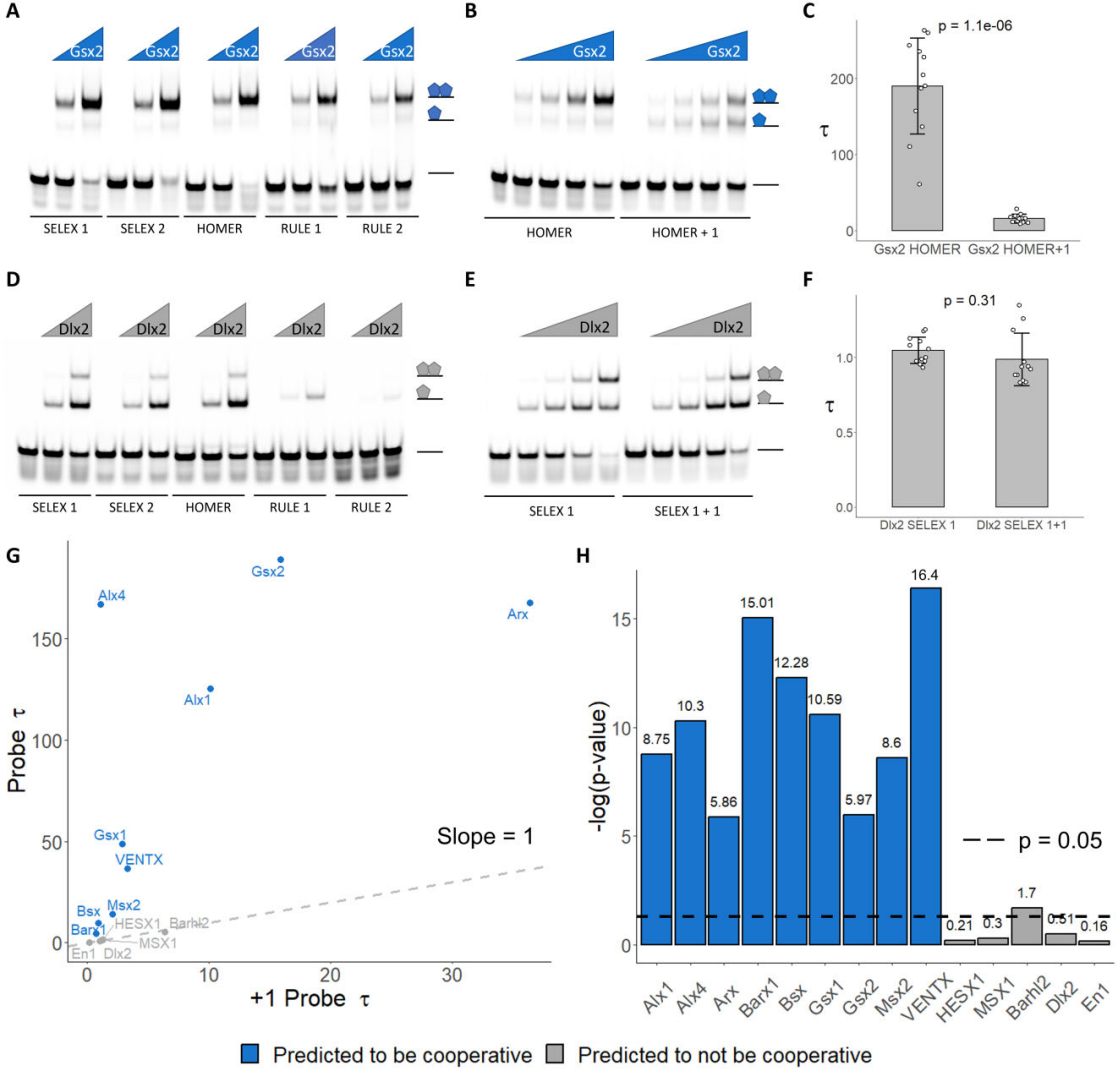
Spacer-dependent cooperativity systematically assessed via EMSAs. (**A**, **D**) The binding activity of five fluorescently labeled probes (34 nM) to two Gsx2 protein concentrations (A; 100 and 400 nM) or Dlx2 protein concentrations (D; 20 and 80 nM) was determined using EMSAs. Schematics at right represent protein-DNA complexes. Note, Gsx2 preferentially binds all probes as a dimer whereas Dlx2 preferentially binds all probes as a monomer. (**B**, **E**) The highest affinity probe for Gsx2 (HOMER) and Dlx2 (SELEX 1) were subsequently used to calculate a cooperativity factor for each TF. To assess for spacer length dependence, we also tested Gsx2 on the HOMER + 1 and Dlx2 on the SELEX 1 + 1 probes in which a single additional bp was added between the 4mers. Each EMSA consisted of 30 lanes in which the 2 probes were tested at 4 concentrations in triplicate. A single replicate is shown here, and all replicates are shown in Supplementary Figure S7. 34 nM of probe was used in each lane and the protein concentrations tested were as follows (B; Gsx2 = 0, 31.25, 62.5, 125, and 250 nM) and (E; Dlx2 = 0, 20, 40, 80 and 160 nM). Schematics of the protein-DNA complexes are shown to the right of each gel. (**C**, **F**) Tau cooperativity factors were calculated for each binding reaction in which the TF was added. Bar graphs depict the average Tau factor for each TF with each dot representing a Tau factor from an individual binding reaction (*n* = 12 for each group). Error bars denote standard deviation. Tau cooperativity factors were compared with two-sided unpaired student t-tests. Note, Gsx2 has a Tau of 189 on the Homer probe, indicating that the binding of the first site facilitates the binding of the second site 189-fold. However, the addition of the nucleotide to the spacer disrupted cooperativity and caused a significant decrease in Tau. In contrast, Dlx2 has a Tau factor of ∼1 on both probes, indicating that the binding of the first site has no impact on the binding of the second site to either probe. (**G**) This approach was applied to 9 TFs that were predicted to bind cooperatively (blue text) and 5 TFs that were not predicted to bind cooperatively (gray text). TFs that were predicted to bind cooperatively had significantly higher Tau factors on their respective high affinity probes (Probe Tau) compared to the probes in which a single bp was added between the two sites (+1 Probe Tau), demonstrating spacer dependent cooperativity. Conversely, TFs that were not predicted to bind cooperatively had similar Tau values between the two probes. Data points that fall on the gray dashed line have equal Tau factors on both probes. (**H**) Bar graph comparing the log_10_*P*-values of the Tau values for the high affinity compared to the +1 probes for each TF. *P*-values that are higher than the dashed line indicate significance. TFs predicted to bind cooperatively had significantly different cooperativities between the two probes, whereas predicted non-cooperative TFs did not with the exception of Barhl2. Note, however, that Barhl2 was weakly cooperative to both probes and had a significantly higher Tau factor for its +1 probe compared to its high affinity probe, demonstrating spacer independent cooperativity.

To test the accuracy of the Cooperativity Predictor, we assessed TF spacer-dependent cooperativity using purified proteins and EMSAs. Since TF cooperativity can be masked by affinity and affinity can be heavily dependent on the flanking and spacer sequences (44), we used a systematic approach to select five different DNA probes for each HD to increase the likelihood that TF cooperativity is accurately assessed in EMSAs. These probes were designed using the following criteria: Two probes were selected based upon the most frequent random 20–30mers in the fourth cycle of the TF’s HT-SELEX assay, labeled SELEX 1 and SELEX 2. One probe was designed to represent the optimal sequence based upon the highest sequence information content at each position across the PWM identified by Homer, labeled HOMER. Lastly, two probes were designed to contain only the core 4mer sequences separated at the ideal distance with all other sequences in the probe being randomly generated GC nucleotides to avoid making additional AT-rich HD binding sites. These two probes are labeled as RULE 1 and RULE 2. Schematics of the probe design are presented in Supplementary Figure S5. Using purified Gsx2 as an example, we found that Gsx2 preferentially bound as a dimer to all designed probes (Figure 3A). However, additional flanking sequences are likely to greatly contribute to Gsx2-DNA binding, as Gsx2 did not bind as well to the RULE 1 and RULE 2 probes that contained appropriately spaced TAAT sequences flanked solely by GC nucleotides (Figure 3A).

To provide a quantitative assessment of HD dimer binding, we next selected the probe with the highest affinity (i.e. for Gsx2, the Homer probe had the highest percentage of depleted free probe when tested at the same protein concentrations) to calculate a cooperativity factor, Tau. Tau is the multiplier that defines how much the binding of a single site facilitates the binding of the second site (see Methods and (15)). A Tau multiplier of 1 would indicate independent, non-cooperative binding, whereas a Tau multiplier >1 would indicate cooperative DNA binding. To determine if cooperativity was spacer specific, we also tested a probe in which a single nucleotide (G) was added in the center of the spacer of the selected probe and called this the +1 probe. If the TF bound cooperatively in a spacer-dependent manner, then the addition of a single nucleotide should disrupt cooperative binding to the probe, and the Tau factor for the selected probe versus the +1 probe should be statistically significant. For example, the optimal high-affinity HOMER probe (TAAT7NTAAT) strongly favored the binding of two Gsx2 proteins, whereas the HOMER + 1 (TAAT8NTAAT) probe was largely bound in an additive manner (Figure 3B). We performed these EMSAs in triplicate and calculated the Gsx2 Tau factor for each probe and found that the rate at which the second binding site of the HOMER +1 probe was being filled was significantly lower than the rate of the second site of the HOMER probe (Figure 3B, C; *P* = 1.06E−06). We next performed the same test using DLX2, a HD factor that was not predicted to have a cooperative dimer site. Similar as Gsx2, Dlx2 bound poorly to the RULE 1 and 2 probes, suggesting that flanking sequences significantly impact Dlx2 binding. However, as predicted, Dlx2 did not bind cooperatively to any of the probes, and the spacer length had no significant impact on the binding characteristics of Dlx2 to DNA (Figure 3D–F; *P* = 0.31), consistent with independent monomeric binding to each site.

### Cooperativity predictor accurately classified homeodomains’ cooperativity capabilities

To assess the accuracy of the Cooperativity Predictor more broadly, we tested a total of 14 TFs for cooperativity using EMSAs. Of these, 9 passed all cooperativity criteria, whereas 5 did not have a predicted cooperative dimer site. As mentioned, the Paired-like Alx1 and Alx4 (9,40) factors and the HOX-like Gsx2 factor (25) have been previously confirmed to cooperatively bind their respective predicted dimer sites. We purified and tested these proteins in EMSAs to ensure that our biochemical methods produced results that were consistent with past work. In addition, we tested the Gsx1 HOX-like factor; the Bsx, Barx1, Msx2 and VENTX NK-like factors; and the Arx Paired-like factor that were all predicted to bind cooperatively. Importantly, comparative analysis of the EMSAs (Supplementary Figure S6; Supplementary Figure S7) revealed that all the predicted cooperative HD TFs had strong spacer length preferences as evidenced by their significantly higher Tau values for their high affinity dimer probe compared to the +1 probe (Figure 3G–H). However, they did differ in their strength of cooperativity as the Paired-like Arx, Alx1, and Alx4 factors as well as the Gsx2 HOX-like factor were highly cooperative (Tau > 100), whereas the Bsx and Barx1 NK-like factors were less cooperative (Tau < 10) (Figure 3G).

As a control for our analysis, we similarly tested five HD TFs (Barhl2, Dlx2, En1, MSX1 and HESX1) that were not predicted to bind DNA in a cooperative manner. Intriguingly, in a prior study, a multinomial method detected dimer sites for MSX1, HESX1 and EN1 (11). However, these dimer sites did not pass the absolute site content and/or information content variability thresholds established by the Cooperativity Predictor algorithm. Importantly, our EMSA and Tau value analysis confirmed these predictions as none of these TFs cooperatively bound their dimer sites in a spacer-dependent manner. The Tau factors of these TFs on their high affinity probes were all approximately 1, consistent with non-cooperative binding (Figure 3G). Further, the Tau factors between the probes of different spacer lengths were not statistically significant (Figure 3H). In contrast, the Tau factors of the TFs predicted to be cooperative were significantly greater than those of TFs not predicted to be cooperative on their respective dimer sites (*P* = 0.011), whereas there was not a significant difference between the calculated Tau values of the +1 probes of these groups (*P* = 0.194). The only unexpected result was obtained for Barhl2, which was not predicted to be cooperative, but had a small, statistically significant increase in Tau on the +1 probe (6.4 ± 1.2) relative to its predicted high affinity probe (5.4 ± 0.6) (Figure 3H; Supplementary Table S8; Supplementary Figure S7L). This finding suggests that Barhl2 has relatively weak cooperativity on DNA, but without a binding preference for a specific spacer length between sites. In total, these findings suggest that the Cooperativity Predictor was able to find previously undetected dimer sites in the case of GSX1, GSX2 and BSX and was able to discriminate between cooperative versus non-cooperative sites with increased accuracy in the cases of MSX1, HESX1 and En1. Moreover, these findings demonstrate that the Cooperativity Predictor both (i) accurately predicted whether a TF could bind cooperatively and (ii) identified specific DNA sequences that facilitated this cooperative binding.

### Cooperativity predictions in the paired-like family correlate with the presence of key amino acids

**Figure 4.**
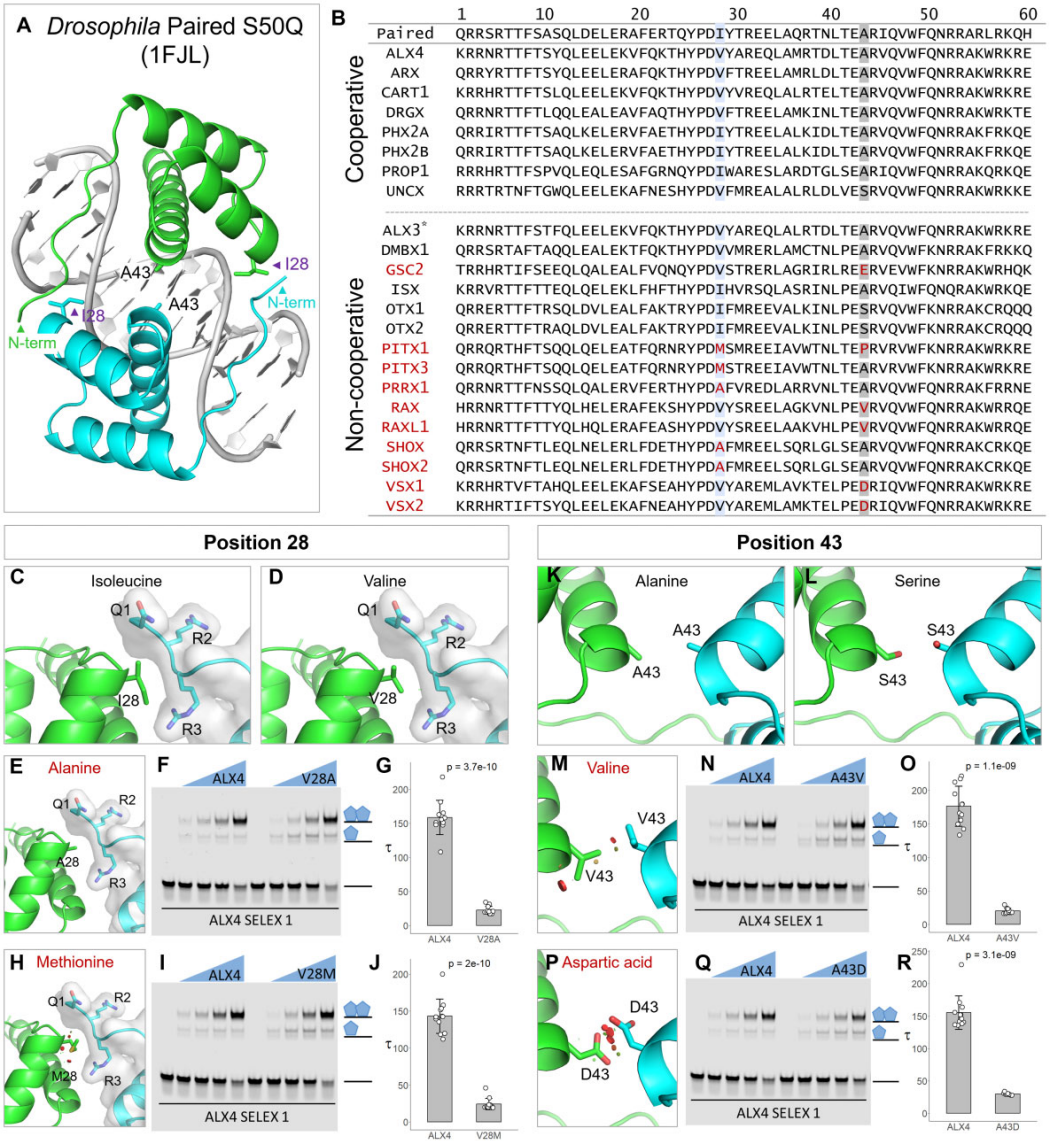
Cooperativity predictions in the Paired-like family are consistent with key amino acids contributing to HD facilitated cooperativity. (**A**) The authors of Wilson et al found that residues 28 and 43 interact at the protein-protein interfaces to facilitate the cooperativity of two *Drosophila* paired HDs on palindromic sites separated by a 3 bp spacer (37). I28 packs with the N-terminal ARM, whereas A43 on both HDs form a symmetrical hydrophobic interaction. (**B**) Sequence alignment of the HDs from cooperative (top) versus non-cooperative (bottom) paired-like TFs demonstrates high variability between the predicted cooperative and non-cooperative TFs in positions 28 and 43. (**C**,**D**) An isoleucine and valine at position 28 can pack against and interact with residues 1 through 3 of the N-terminal ARM of the second HD. (**E**) An alanine at position 28 is predicted to make fewer nonpolar interactions. (**F, G**) To test the influence of this residue on cooperativity, we performed EMSAs using a purified ALX4 protein containing a V28A mutation. An ALX4 residue switch from valine to alanine at position 28 reduced the protein's cooperativity 7-fold. Note, each EMSA experiment in this Figure consisted of 30 lanes in which two proteins (WT and the variant of interest) were tested at 4 concentrations in triplicate. A single replicate is shown here, and all replicates are shown in Supplementary Figure S8A-D. 34 nM of probe was used in each lane and the protein concentrations tested were 0, 18.75, 37.5, 75 and 150 nM. Schematics of the protein-DNA complexes are shown to the right of each gel. Tau cooperativity factors were calculated for each binding reaction in which the TF was added. Bar graphs depict the average Tau cooperativity factor for each TF with each dot representing a Tau factor from an individual binding reaction (*n* = 12 for each group). Error bars denote standard deviation. Tau cooperativity factors were compared with two-sided unpaired Student *t*-tests. (**H**) A methionine at position 28, as in PITX, produces van der Waals overlap that would cause clashing at this interface (red discs). (**I, J**) An ALX4 valine to methionine mutation at position 28 reduced the cooperativity of ALX4 6-fold. (**K**) Alanine residues at position 43 in the Paired structure form a symmetrical hydrophobic interaction. (**L, M, P**) A serine at position 43 as found in OTX can also be accommodated between the two HD proteins, however a valine as seen in the RAX factors or aspartic acid as seen in the VSX factors at position 43 is predicted to cause considerable clashing between residues as shown by the red discs. (**N, O, Q, R**) Quantitative EMSAs showed that altering the ALX4 residues from A43V or A43D decreased cooperativity ∼9 and 5-fold respectively. *Note, although HT-SELEX performed on ALX3 did not enrich for the palindromic dimer site and the factor was not predicted to bind cooperatively by the Cooperativity Predictor, it should be noted that Alx3 has been shown in EMSAs to bind cooperatively to the Paired-like site in past studies (65).

Analysis of the Paired-like subclass of HDs revealed that over a third of these TFs are predicted to bind cooperatively to highly similar, if not identical, palindromic sites 3 bp apart (Figure 2, bottom right). We next wanted to determine if this cooperativity was related to specific amino acids that were conserved in the predicted cooperative TFs but not the non-cooperative TFs. To take a targeted approach, we took advantage of information derived from the crystal structure of a S50Q variant *Drosophila* Paired HD bound as a cooperative dimer on the TAAT3NATTA palindromic site (37) (Figure 4A). The authors identified specific interactions between residues within the 60 amino acid Paired HD that facilitated cooperativity: (i) E42 was found to form a water mediated intermolecular hydrogen bond with R44 and an intermolecular hydrogen bond with R3; (ii) I28 of one HD fits tightly against the N-terminal ARM of the second HD (Figure 4A) and (iii) A43 creates symmetrical hydrophobic interactions between the two HDs (37) (Figure 4A). Alignment of the predicted cooperative versus non-cooperative Paired-like factors revealed that R3, E42, and R44 are conserved across all paired-like factors, however the 28th and 43rd residues vary between the paired-like factors, possibly signifying that these residues may convey cooperative versus non-cooperative activity (Figure 4B).

To assess the impact of the different residues at the 28th and 43rd positions, we first modeled residue changes present in the cooperative and non-cooperative Paired-like factors using Pymol, and then tested a subset of these predictions within the highly cooperative ALX4 protein using EMSAs and Tau factor calculations. At position 28, the branched nonpolar amino acids, valine and isoleucine, are found in all the predicted cooperative TFs (Figure 4B–D). However, alanine, which is predicted to not make similar favorable nonpolar interactions is found in three of the non-cooperative TFs (Figure 4E). In agreement with these predictions, we found that changing the ALX4 valine to alanine in the 28th position reduced cooperativity ∼7-fold (Figure 4F–G). Moreover, a methionine at position 28, as seen in the PITX factors, is predicted to be unable to tightly pack with the N-terminal ARM due to van der Waals overlap (red disks) (Figure 4H), and we found that a V28M variant in ALX4 negatively impacted cooperativity ∼6-fold (Figure 4I–J). Next, we modeled the different residues found at position 43, which forms a hydrophobic interaction at the protein-protein interface between the HDs in the *Drosophila* Paired structure (37). The alanine and serine residues found in cooperative factors possess side chains that fit within this 4.2 angstrom gap without steric clashes (Figure 4K–L). However, valine and aspartic acid residues in this position, which are found in the non-cooperative, RAX and VSX Paired-like TFs, would be unable to fit due to clashing between amino acids (Figure 4M, P). Experimentally, we found that ALX4 proteins with the A43V and A43D mutations reduced cooperativity ∼9- and ∼5-fold, respectively (Figure 4M–R). Similarly, proline and glutamic acid at the 43^rd^ positions as seen in PITX1 and GSC2 are also predicted to reduce cooperativity due to steric clash at this protein–protein interface (Supplementary Figure S8I-J). Importantly, these residue changes did not dramatically affect DNA binding affinity, as all protein variants had similar free (unbound) probe depletion (Supplementary Figure S8A–H).

While variants in the 28th and 43rd positions provide insight into why the RAX, SHOX, GSC2, PITX and VSX factors fail to enrich for the palindromic dimer site, these two positions alone fail to provide a structural explanation for five of the predicted non-cooperative Paired-like factors (Figure 4B). Further, these residue changes only influenced the magnitude of cooperativity within the ALX4 protein and did not abolish the ability of this protein to bind in a cooperative manner. To ensure that the Cooperativity Predictor algorithm accurately classified the TFs in which these required residues were present, we tested Isx, one of the predicted non-cooperative factors that has the preferred I28 and A43 residues found in cooperative factors, on an ideal Paired-like TAAT3NATTA site in EMSA. As expected, based on the Cooperativity Predictor output, Isx was unable to bind cooperatively to this DNA site (Supplementary Figure S8K), suggesting that additional residues influence the cooperativity of these factors. In summary, we found that residues at the 28th and 43rd positions can clearly contribute to cooperative DNA binding, but further studies will be needed to determine the other residues within the Paired-like family that are required for cooperative complex formation on DNA.

### Homeodomains bind cooperative dimer sites *in vivo*

We next sought to assess the *in vivo* prevalence of the predicted cooperative dimer sites using available genomic binding data (ChIP-seq and CUT&RUN) for the Gsx2, PHOX2B, Phox2a, MSX2 and Barx1 TFs through the following general strategy: First, we separated the called peaks into three categories: peaks that contained at least one HT-SELEX dimer site, peaks that contained at least one HT-SELEX 4mer but not a dimer site, and peaks that contained neither site. Second, we plotted the motif densities of the dimer-dependent and dimer-independent sites for each category to confirm motif enrichment at the peak centers. Third, we plotted the fold changes of the read strengths to compare the binding signals between these categories of peaks. Fourth, we applied COSMO on the called peaks to determine if a dimer site consisting of the 4mers separated at the identified spacer length found in HT-SELEX was also specifically enriched in the genomic binding data.

**Figure 5.**
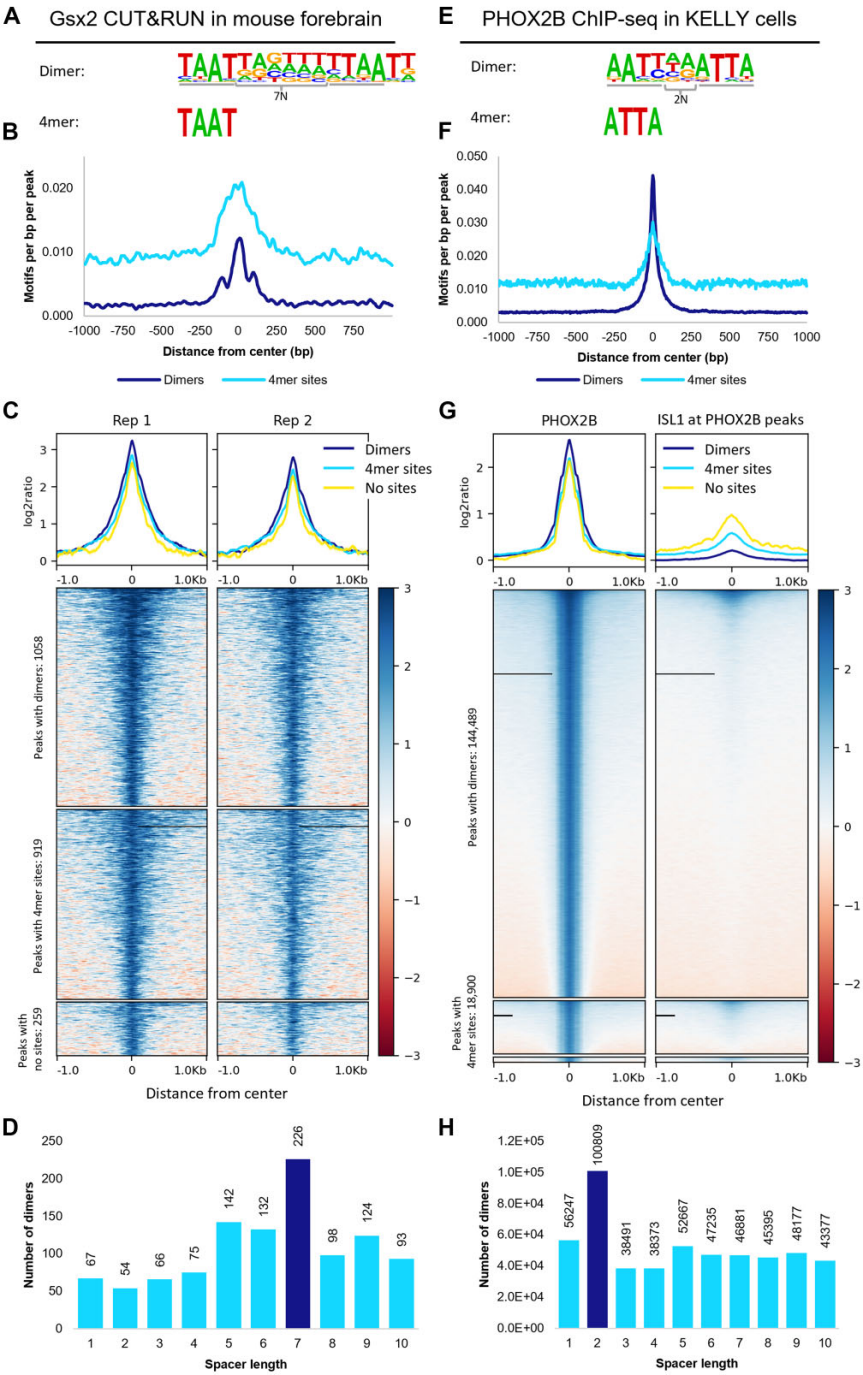
Genomic binding assays reveal that predicted TFs bind cooperative dimer sites and non-cooperative monomer sites *in vivo*. (**A**) HT-SELEX revealed that GSX2 binds to a dimer site with TAAT 4mers spaced 7 bp apart. (**B**) Dimer motifs and the identified 4mers are highly prevalent in the centers of peaks called from mouse forebrain CUT&RUN assays. (**C**) Peaks containing Gsx2 dimer sites had a higher binding signal than peaks containing only 4mer sites or neither site, consistent with dimer sites either being bound more frequently or with higher stability than monomer sites. (**D**) Dimer sites consisting of 4mers 7bp apart are highly enriched compared to 4mers spaced by alternate distances. (**E**) PHOX2B HT-SELEX data enriched for a dimer motif consisting of 4mers 2bp apart and a monomer ATTA site. (**F**) These motifs are highly prevalent at the peak centers and **(G)** peaks containing a dimer site have increased binding compared to peaks with only a monomer site or lacking both types of sites. ISL1 does not bind many of the PHOX2B bound regions, which is consistent with these dimer sites being bound by PHOX2B homodimers rather than PHOX2B-ISL1 heterodimers. (**H**) 4mers spaced 2bp apart occurred nearly twice as frequently as the next most frequent spacing, consistent with the spacer specificity seen in cooperativity.

We first validated this approach by applying it to Gsx2 mouse forebrain CUT&RUN data, in which Gsx2 was previously shown to bind both cooperative and non-cooperative sites *in vivo* (25). Analysis of the dimer and 4mer motifs revealed each was highly enriched at the peak centers for each respective category, consistent with Gsx2 binding both types of sequences (Figure 5B). Moreover, the binding signal of the peaks with dimer sites was slightly higher than those peaks with only 4mer sites (Figure 5C), suggesting that dimer sites are more frequently bound and/or that the interactions between two proteins improve the stability of the Gsx2-DNA complex. Further, the category of peaks that did not contain either site type was the smallest and had the lowest binding signal (Figure 5C), consistent with Gsx2 requiring HD sites to bind and regulate target genes. Lastly, COSMO analysis of the peaks revealed that dimer sites spaced 7 bp apart occurred more frequently than those at alternative spacer lengths (Figure 5D), consistent with the findings from the HT-SELEX analysis (Figure 1E). Hence, this analysis is consistent with past work that showed Gsx2 binds both cooperative and non-cooperative sites *in vivo* (25).

We similarly analyzed available data for the following other cooperative TFs: PHOX2B ChIP-seq from the KELLY (45), BE2C (45) and CLB-GA (46) neuroblastoma cell lines; Phox2a ChIP-seq from mouse induced cortical motor neurons (47); MSX2 ChIP-seq from human trophoblast stem cells (48); eGFP-MSX2 ChIP-seq from the MCF7 cell line (49); and FLAG ChIP-seq performed on 3xFLAG-Barx1-EGFP infected immortalized E13.5 stomach mesenchymal cells (50). However, three of the datasets were difficult to interpret for different reasons: the Phox2a study in differentiated motor neurons co-expressed the Isl1 TF using doxycycline prior to ChIP-seq, making it difficult to distinguish between Phox2a homodimerization versus Phox2a/Isl1 heterodimerization (Supplementary Figure S9I-K), whereas the two MSX2 datasets had low overall motif enrichment in called peaks (Supplementary Figure S10A–H). In contrast, all the other *in vivo* datasets revealed significant enrichment for both the predicted monomer and dimer sites identified by the HT-SELEX data analysis. Comparative peak analysis largely revealed higher binding signal in peaks with dimer sites versus those that lacked dimer sites with the exception of BARX1 (Figure 5; Supplementary Figure S9; Supplementary Figure S10). For example, the PHOX2B datasets provided clear evidence for both cooperative and non-cooperative sites *in vivo* with dimer and monomer site motifs heavily enriched in the center of most peaks called from PHOX2B ChIP performed on the KELLY cell line (Figure 5E). In fact, ∼87% of the called peaks contained a dimer site and only ∼1% did not contain either site type, and those peaks with dimer sites had a higher binding signal than peaks with only monomer sites or peaks that lacked either type of site (Figure 5G). As mentioned above, PHOX2A, a close homologue to PHOX2B, can heterodimerize with ISL1 on a similar TAAT3NATTA motif (47). Hence, to ensure that the increased binding signal and motif localization was not due to heterodimerization, we plotted the ISL1 ChIP-seq signal on the PHOX2B peaks (Figure 5G). This analysis revealed very little co-localization between these factors in this cell line, suggesting that these findings are independent of ISL1 heterodimerization and increasing the likelihood that the palindromic motif is enriched due to PHOX2B homodimerization. Further, COSMO analysis showed that a dimer site with 4mers 2bp apart (note, the PHOX2B dimer motif (AATT2NATTA) identified by the Cooperativity Predictor is shifted by a single bp relative to the TAAT3NATTA motif (Supplementary Figure S4B)) was highly enriched, which is again in agreement with the results found in HT-SELEX (Figure 5H). Importantly, similar results were found across all three neuroblastoma cell lines (Supplementary Figure S9). Thus, these *in vivo* analyses are consistent with our *in vitro* predictions from the HT-SELEX data, and thereby provide further evidence that a subset of HD TFs utilize cooperative homodimer formation to enhance DNA binding specificity to AT-rich DNA sequences by binding sites of distinct spacer lengths. Supplementary Table S9 provides a summary of the computational predictions, EMSA results, and *in vivo* analyses results.

## DISCUSSION

In this study, we evaluated the prevalence of cooperativity in the HD family. First, we designed the computational pipeline, Cooperativity Predictor, to identify cooperative HD TFs and their DNA dimer sites using only HT-SELEX data. Out of the 88 human and 24 mouse HDs analyzed, we predicted 15 human and 7 mouse HDs to exhibit cooperative behavior. We next used quantitative EMSAs to calculate the cooperativity of 14 HDs on DNA, 9 of which were predicted to be cooperative and 5 of which were not predicted to bind in a cooperative manner. All but 1 of the tested HDs were correctly characterized by the Cooperativity Predictor: the 9 HD TFs predicted to be cooperative were confirmed to cooperatively bind dimer sites with constrained spacer lengths, whereas 4 of the 5 HD TFs predicted as non-cooperative were confirmed to bind in a non-cooperative manner. Moreover, the one exception (Barhl2) was weakly cooperative on both the identified dimer site and the +1 probe, and thus the spacer-independent binding behavior of Barhl2 to DNA explains why it was not detected by the Cooperativity Predictor pipeline. Importantly, 6 of the confirmed cooperative TFs had not been previously shown to exhibit cooperative behavior to our knowledge. We subsequently used structural models and amino acid conservation to highlight the roles of two key HD positions that are likely to help discriminate between cooperative versus non-cooperative Paired-like HDs. Lastly, we demonstrated that the Gsx2 and PHOX2B factors bind to their predicted cooperative dimer sites *in vivo* through the analysis of available genomic data. Below, we highlight two key implications of these findings: First, we discuss the general utility of using HT-SELEX data and the Cooperativity Predictor to identify cooperative DNA binding sites. Second, we describe how the differential formation of cooperative HD dimers on binding sites with distinct spacing requirements can greatly enhance HD TF DNA specificity.

### Streamlined approach to characterize HD cooperativity

Unbiased *in vitro* DNA binding assays such as HT-SELEX and PBMs followed by motif search algorithms have revolutionized our ability to systematically define TF binding sites. In the original HT-SELEX study, Jolma et al processed the HT-SELEX data to identify the most enriched 6–12 bp subsequences and then applied a multinomial method to generate models that incorporate dinucleotide base biases. Importantly, their studies revealed that a subset of TFs not only enriched for monomer sites, but also for dimer sites that suggested formation of homodimer complexes on specific DNA sequences (11). Since this data and analysis were released in 2013, multiple other approaches have been applied, ranging from machine learning (24,51–53), deep learning (54) and complex algorithms (55–57). These approaches have typically focused on better defining monomer binding specificities or known dimer complex specificities such as those from the bZIP or bHLH families. Computational analyses that focused on cooperativity have investigated heterodimer cooperativity using both HT-SELEX (11) and consecutive-affinity-purification SELEX (CAP-SELEX) data (58). For example, Rube *et al.* developed a three-layer maximum likelihood model, ProBound, that combines distinct DNA binding assays to develop comprehensive TF binding models and quantify absolute binding affinities. The authors applied ProBound to discover cooperative binding configurations and cooperativity contributions of individual factors through the analysis of CAP-SELEX and HT-SELEX datasets (24). Furthermore, Ibarra et al generated models to incorporate DNA shape, dinucleotide dependencies, and DNA sequence preference to predict the heterodimeric cooperativity of FOXO1 and ETS1 (59). They subsequently integrated the derived cooperative kmers with ChIP-seq and ontology data to find a relationship between FOXO1-ETV6 cooperativity and chronic lymphocytic leukemia, successfully linking cooperativity to a disease state (59). It is important to note however, that these analyses also require a CAP-SELEX dataset to have been performed on the same TF with two different tags in the same experiment to assess homodimeric cooperativity. Out of the HDs analyzed here, the required HT-SELEX and CAP-SELEX datasets are only available for ALX4 and HOXB13. Therefore, Cooperativity Predictor distinguishes itself by assessing cooperativity using only HT-SELEX data.

Other studies have used datasets outside of HT-SELEX to identify dimer binding sites such as chromatin accessibility data (60), genomic binding assays (61,62), gene expression data (63), and transcriptional output assays (64). However, it is often difficult to differentiate between homodimer binding versus heterodimer binding in genomic assays with low resolution as seen in our analysis of the Phox2a ChIP-seq in differentiated motor neurons (Supplementary Figure S9I–L). To circumvent this problem, a recent study developed a convolutional neural network on a genomic assay with single bp resolution, ChIP-nexus, to define the cooperativity syntax of four TFs (62). With the increased resolution, BPNet could differentiate between heterodimeric and homodimeric binding, and the authors were able to identify spacer length dependent protein-to-protein interactions that were too subtle to be detected by previous methods (62). Unfortunately, there is limited genomic binding data available, especially at the bp resolution recommended for the described method, to broadly apply this approach to many TFs.

Despite these available tools and resources, the field lacked a pipeline that identifies homodimer cooperative interactions in a streamlined manner from an assay with abundant available datasets. To address this need, we developed a computational pipeline with several methodological features optimized to identify HD TFs that exhibit cooperative behavior by tailoring already available bioinformatics tools and applying statistical tests specific to HT-SELEX data. First, we processed the data from all SELEX cycles as a single experiment, allowing us to use motif enrichment through the SELEX cycles as a measure of cooperativity. Second, as dimer sites tend to range between 12 and 18 bp in length, we specifically searched for longer enriched motifs (16 and 18 bp). Third, and most importantly, we employed two approaches based on independent ideas of cooperativity: (i) We compared the enrichment rates of dimer versus independent 4mers through the SELEX cycles as a predictor of cooperativity based on the idea that cooperative complexes are more stable on DNA than monomer TF-DNA complexes. (ii) We assessed the specificity of spacer lengths between the top 4mer sequences because cooperative interactions typically require dimer sites with a specific spacer length. Through this approach, we successfully identified several new cooperative HDs and dimer binding motifs from only HT-SELEX data, several of which were not predicted to bind dimer sites by other computational methods. Thus, in contrast to other more complex search algorithms that often focus on heterodimeric interactions, the Cooperativity Predictor algorithm accurately identifies homodimer interactions on constrained DNA binding sites using abundant available datasets from the HT-SELEX assay.

While we specifically designed the Cooperativity Predictor algorithm for HD TFs, future studies could focus on optimizing the algorithm to predict cooperative DNA binding for other TF families by modifying the site and spacer length requirements based on biochemical and structural data. For HD TFs, we used a 4mer length based on prior structural data that revealed HD proteins make key contacts with a 4bp core sequence (i.e. TAAT) (8). However, the length of the site could be shortened or extended based upon known DNA binding sequence requirements for each TF family. In addition, since 4mer sites are relatively short, we required the two 4mers to be at least 2 bp apart for HD TFs to help distinguish between long monomer sites versus dimer sites. This 2 bp spacer requirement means that the Cooperativity Predictor will fail to identify HD TFs that bind dimer sites without a spacer or only a 1 bp spacer. However, sites separated by 0 and 1 bp spacers have a total length of 8 and 9 bp, which would likely be detected using standard search algorithms such as the IniMotif subsequence search used by Jolma *et al.* (11,23). For other TF families, the spacer lengths between sites could be adjusted depending upon the expected differences in motif lengths of monomer versus dimer sites. As the Cooperativity Predictor classifies a dimer site using spacer information content and length, dimer sites with spacers that contain high information content or have flexible lengths will not be detected by the pipeline. Hence, the Cooperativity Predictor pipeline may underestimate the number of HD TFs capable of cooperative DNA binding. For example, we found that Barhl2, which was predicted to be non-cooperative, has weak cooperativity to dimer sites with flexible spacer lengths. Lastly, it should be noted that the dimer versus monomer motif enrichment module of the Cooperativity Predictor requires the TF to bind as both a monomer and dimer. We did not find this to be a significant limitation for analyzing HD factors because each was found to enrich for monomer sites. However, if the TF is found to only bind DNA as a dimer, the COSMO module could be used to assess for distinct spacer lengths between binding sites.

### HD cooperativity impact on DNA binding specificity

The HD family is one of the largest groups of TFs in metazoans, and HD factors regulate diverse developmental and homeostatic processes. Defining how proteins that bind highly similar DNA sequences *in vitro* regulate different specialized processes has been a long-standing problem in the field of transcriptional regulation. Increasingly, studies have revealed that HD TFs increase their DNA binding specificity by forming homo- and heterodimer complexes on DNA. The requirement of two proteins binding distinct sites separated by a specific distance and orientation lengthens the DNA recognition sequence, and thereby increases TF-DNA specificity via two mechanisms. (i) The likelihood of finding two sites at a specific spacer length at random is lower than the likelihood of finding a single site. This makes the TF’s binding sites more specific and targeted. (ii) The enhanced cooperativity of a TF complex reduces the number of TFs that can effectively compete for sites as only specific TFs cooperatively bind such dimer sites.

In our study, we identified several new HDs that are capable of cooperatively binding on specific HD dimer motifs in a manner that increases DNA binding specificity. For example, while all 88 members of the HD family analyzed here bind highly similar AT rich monomer sequences, only BSX was able to cooperatively bind AT rich sites 4bp apart and only VENTX was able to cooperatively bind AT rich sites 9 bp apart (Figure 2). Hence, BSX and VENTX would be predicted to have a selective advantage over other HD TFs to bind targets containing their respective dimer motifs. Further comparisons between dimer motifs reveals that in addition to spacer length differences, binding site orientation also differs between these motifs. For example, Gsx2 binds in a head-to-tail orientation (25), whereas the Paired crystal structure showed that this complex bound in a head-to-head orientation (37). Lastly, our search algorithm both confirmed and expanded the number of Paired-like HDs capable of cooperatively forming homodimers on DNA, as we found that approximately 1/3 of Paired-like HD factors selectively enriched for the TAAT3NATTA palindromic motif (Figure 2; Supplementary Figure S4B). Collectively, these studies highlight how the selective formation of cooperate HD complexes on constrained binding motifs allows for factors to bind diverse recognition sequences despite containing highly similar HD DNA binding domains capable of largely binding to the same monomer sequences.

A question that emerges from our studies is what key HD amino acid residues and/or additional domains are required for the selective formation of cooperative homodimer complexes on DNA? Cooperative DNA binding typically results from additional protein-protein interactions between TFs that supplement the protein to DNA interactions to stabilize the complex on DNA and/or from a DNA conformation change induced by the binding of the first protein that increases stability of binding by the second protein (38,39). For example, the crystal structure of the Paired factor on its preferred dimer site demonstrated that HDs bend DNA and thereby position the adjacent protein so that they physically interact (37). These stabilizing interactions decrease the off-rate, which in turn raises TF-DNA affinity. Using sequence conservation between cooperative and non-cooperative paired-like factors and the existing Paired-DNA homodimer structure as a model, we identified and tested the role of several key residues within the HD that are likely to help explain differences in cooperative versus non-cooperative DNA binding activity (Figure 4). In contrast, Gsx2 likely requires sequences outside the HD for optimal cooperative DNA binding, as prior studies have shown that the Gsx2 HD alone is less cooperative than a Gsx2 protein containing an additional 40 amino acids C-terminal of its HD (25). Less is known about the key amino acid sequences required for the formation of the other cooperative HD complexes. However, it is important to note that the proteins used in this study and in the HT-SELEX assay typically contained 20–30 amino acids flanking the HD. Thus, the cooperativity of these HD factors must be mediated via either direct HD-HD interactions or via nearby flanking residues. These limited regions appear to be drivers of cooperativity in most cases. For example, by comparing the HT-SELEX results between the TFs’ DNA binding domains and full-length proteins, Jolma et al found that the binding specificities were similarly established by the truncated region in 78 of the 79 TFs tested (11). Moreover, we also found that the Cooperativity Predictor predicted like cooperative behavior between the tested shorter HD proteins and the full-length proteins in 19 out of 21 HD TFs (Supplementary Table S10). Thus, homodimeric cooperativity appears to be primarily mediated through the HD and flanking regions. However, future studies using sequence conservation, mutagenesis, and structural approaches will be needed to ascertain the likely diverse mechanisms used by HD factors to mediate cooperative DNA binding.

Lastly, cooperativity has been shown to impact transcriptional output as well as DNA binding specificity. A recent massively parallel reporter assay of the paired-like HD, CRX, revealed that enhancers with dimeric sites produce a stronger signal than those with monomeric sites (16). Moreover, the ALX factors appear to only generate transcriptional changes using dimer sites, in spite these proteins being able to bind monomer sites (Supplementary Figure S7A, B) (9,65). Taken together, these findings suggest that the added DNA binding affinity and/or specificity of CRX and ALX factors for their respective dimer sites results in increased levels of gene expression output. In contrast, prior studies revealed Gsx2 mediates opposing transcriptional outputs in a binding site dependent manner as Gsx2 binding to dimer sites was associated with gene stimulation whereas Gsx2 binding to monomer sites was associated with gene repression (25). Moreover, the authors tested more complex enhancer elements with different ratios of monomer to dimer sites to demonstrate how the higher affinity dimer sites could lead to gene stimulation at low Gsx2 levels, whereas high levels of Gsx2 filled both monomer and dimer sites and led to transcriptional repression (25). These findings highlight how the differential use of cooperative versus non-cooperative sites can result in distinct transcriptional outputs in a TF concentration and binding site dependent manner. Even with these defined mechanisms, the extent of cooperativity's role in gene regulation discovered thus far is most likely understated, and the manner and scope in which these transcriptional changes relate to disease states have yet to be investigated. Here, we evaluated the prevalence of cooperative behavior in the HD family, however further investigation will be needed to understand the extent in which cooperativity is altering DNA binding specificity, affinity, and transcriptional outputs to impact gene regulation.

## DATA AVAILABILITY

Cooperativity Predictor is available at https://github.com/cainbn97/Cooperativity_predictor and https://doi.org/10.5281/zenodo.7254022.

## Supplementary Material

gkad318_Supplemental_Files
