# Supplementary material for: Prediction of cooperative homeodomain DNA binding sites from high-throughput-SELEX data
Source: Nucleic Acids Res. 2023 Apr 28;51(12):6055–72. doi: 10.1093/nar/gkad318 (PMC10325903; doi:10.1093/nar/gkad318)
Supplement: gkad318_Supplemental_Files [file gkad318_supplemental_files.zip › Supplementary_Information_final.pdf]

SUPPLEMENTARY FIGURES

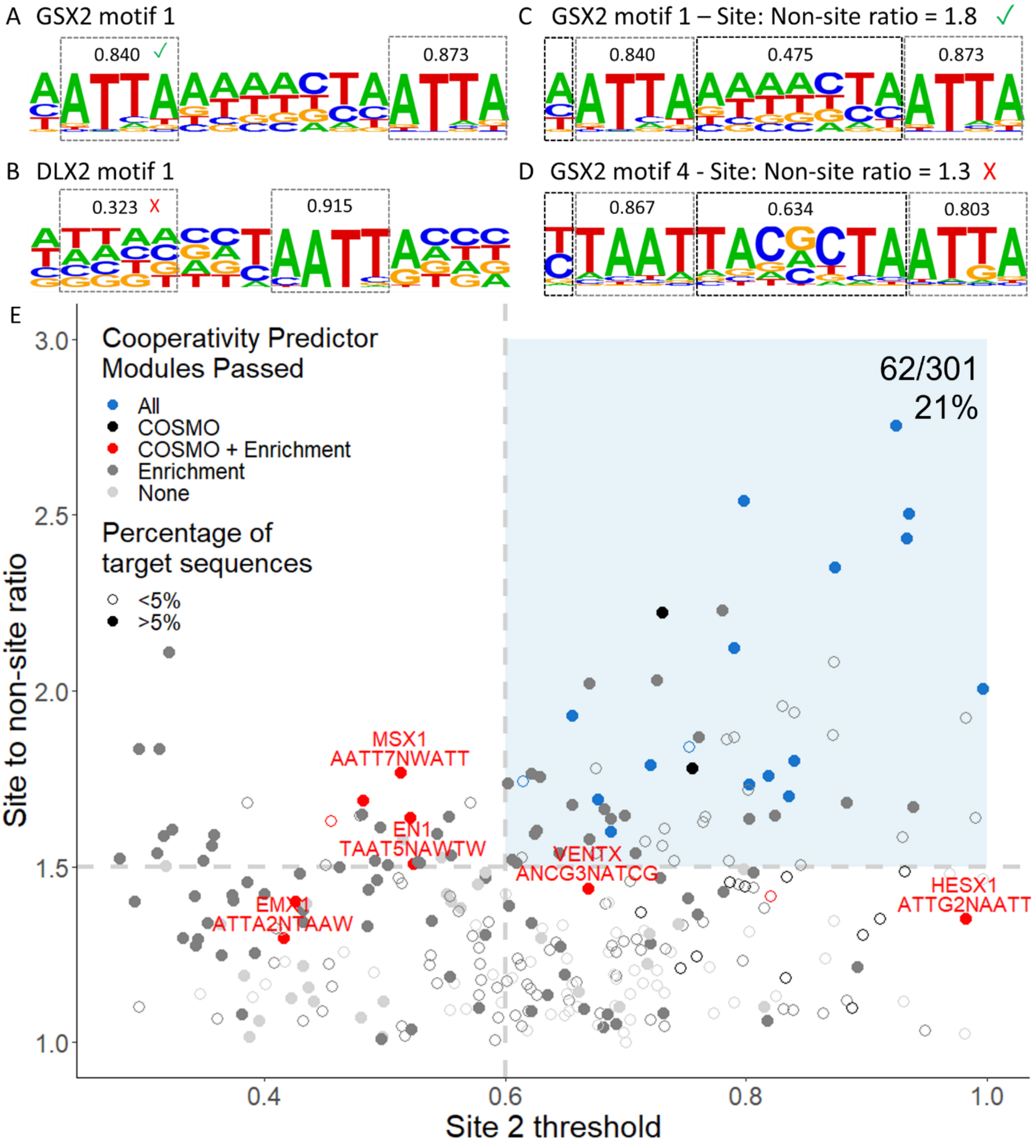

Supplementary Figure 1. Dimer sites were identified from long motifs using the information content of two 4mers and the variation in information content in the full PWM. We applied two filters to differentiate between long monomer sites and true dimer sites as well as to reduce PCR amplification bias. These filters have the added benefit of reducing computational time required for analysis and the chance of false positives. The first filter considers that the 4mer with lower information content (by definition, site 2)

must meet a minimum threshold of information content, and the second filter considers the site-to-non-site ratio threshold that compares the variation of information content between the 4mers and the surrounding regions. To define these filter thresholds, we looked at the entirety of the HD family data and processed all dimer sites found by the Homer *de novo* motif analysis with the Cooperativity Predictor pipeline. The site 2 filter requires that the PWM have two distinct high information content regions, consistent with a dimer site. **(A)** For example, the GSX2 motif 1 PWM has two distinct 4mers with information content greater than 0.6, whereas **(B)** the DLX2 motif 1 has a single site above 0.6 with no surrounding regions displaying high information content, which is consistent with a motif displaying a single site. The site-to-non-site ratio threshold aimed to remove any PWMs with high information content across each position of the motif, which is consistent with replicate 20 and 30mers being amplified through PCR bias rather than true non-duplicate sequence selection consistent with cooperative behavior. **(C)** For example, the top motif for GSX2 (motif 1) had an average site information content of 0.857 and a non-site average information content of 0.475, consistent with the PWM being generated from a relatively complex list of sequences. **(D)** In contrast, GSX2 motif 4 had only a slight difference between the average information content of the top sites and surrounding region, which could be indicative of possible PCR duplicates. **(E)** 301 dimer sites were found across the *de novo* motif analysis results for the 88 HDs. By setting the site 2 threshold to 0.6 and the site to non-site ratio threshold to 1.5 (denoted by the light blue area), we have gated the dimer sites that proceed to dimer to monomer site enrichment and COSMO analyses. This gating drastically reduces computational time to run the pipeline as only 21% of the found dimer sites required post analysis. Further, these thresholds reduce the chance of false positives. The plot displays the number of dimer sites that passed each of the modules of the Cooperativity Predictor: All three modules (blue), the COSMO + Enrichment modules but not the dimer site thresholds (red), COSMO (black), Enrichment (gray), and none (light gray). Whether the dimer site of interest appeared in over 5% of the sequences in the fourth cycle of HT-SELEX is denoted by the fill of datapoints. Note, the number of dimer sites that occur in less than 5% of the target sequences predominantly have a site to non-site ratio of less than 1.5 which is consistent with motifs with low information content variability arising from a few overamplified sequences. There were several HDs that had dimer sites that were predicted to bind cooperatively in the enrichment and COSMO modules (red) but did not meet these dimer site criteria. Three of the TFs that bordered our thresholds were tested biochemically (see Supplementary Figure 6; Supplementary Figure 7) and were found to not bind cooperatively to their respective sites.

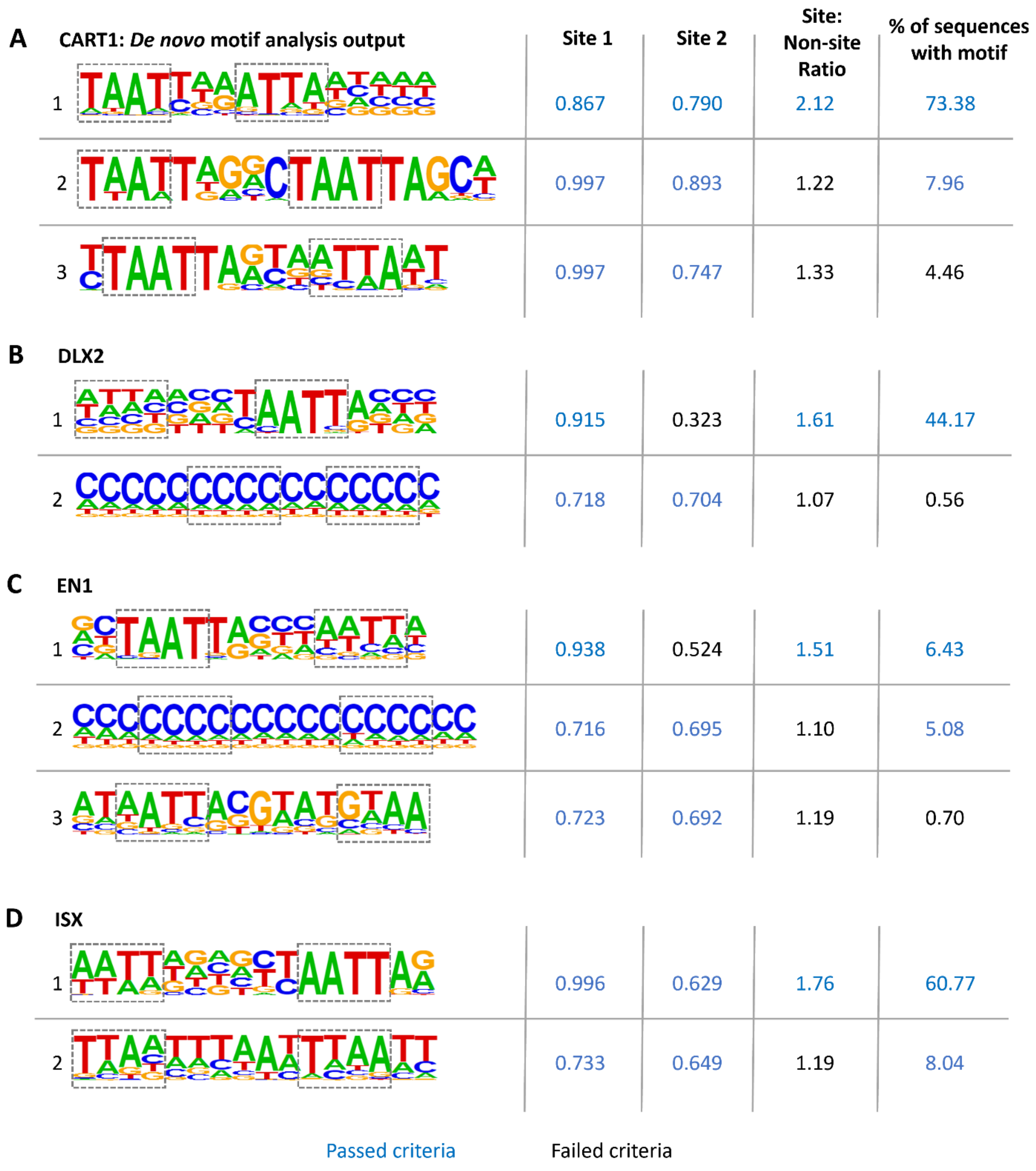

Supplementary Figure 2. Dimer sites were distinguished from monomer sites through the implementation of several thresholds: The average absolute information content across the individual 4mers had to be greater than 0.6, the information content of the selected 4mers had to be 1.5 times greater than the surrounding flanking regions and spacer, and the identified dimer site had to appear in at least 5% of the cycle 4 sequencing pool. Examples of how these thresholds filtered through the *de novo* motifs found by Homer are shown for **(A)** CART1, **(B)** DLX2, **(C)** EN1, and **(D)** ISX.

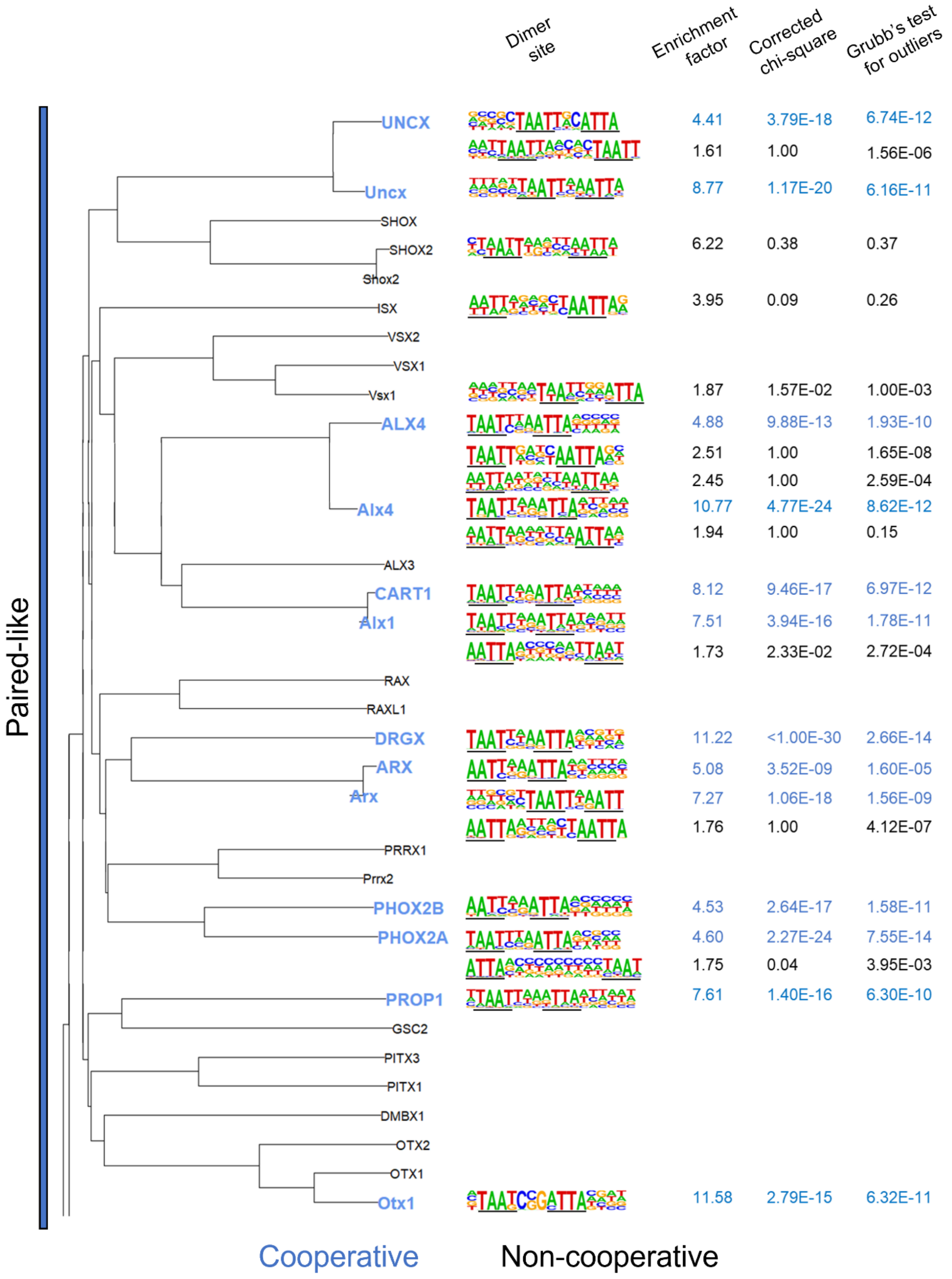
Dimer site
Enrichment factor
Corrected chi-square
Grubb's test for outliers
Paired-like
UNCX 4.41 3.79E-18 6.74E-12
1.61 1.00 1.56E-06
Uncx 8.77 1.17E-20 6.16E-11
SHOX
SHOX2 6.22 0.38 0.37
Shox2
ISX 3.95 0.09 0.26
VSX2
VSX1
Vsx1 1.87 1.57E-02 1.00E-03
ALX4 4.88 9.88E-13 1.93E-10
2.51 1.00 1.65E-08
2.45 1.00 2.59E-04
Alx4 10.77 4.77E-24 8.62E-12
1.94 1.00 0.15
ALX3
CART1 8.12 9.46E-17 6.97E-12
Alx1 7.51 3.94E-16 1.78E-11
1.73 2.33E-02 2.72E-04
RAX
RAXL1
DRGX 11.22 <1.00E-30 2.66E-14
ARX 5.08 3.52E-09 1.60E-05
Arx 7.27 1.06E-18 1.56E-09
1.76 1.00 4.12E-07
PRRX1
Prrx2
PHOX2B 4.53 2.64E-17 1.58E-11
PHOX2A 4.60 2.27E-24 7.55E-14
1.75 0.04 3.95E-03
PROP1 7.61 1.40E-16 6.30E-10
GSC2
PITX3
PITX1
DMBX1
OTX2
OTX1
Otx1 11.58 2.79E-15 6.32E-11
Cooperative
Non-cooperative

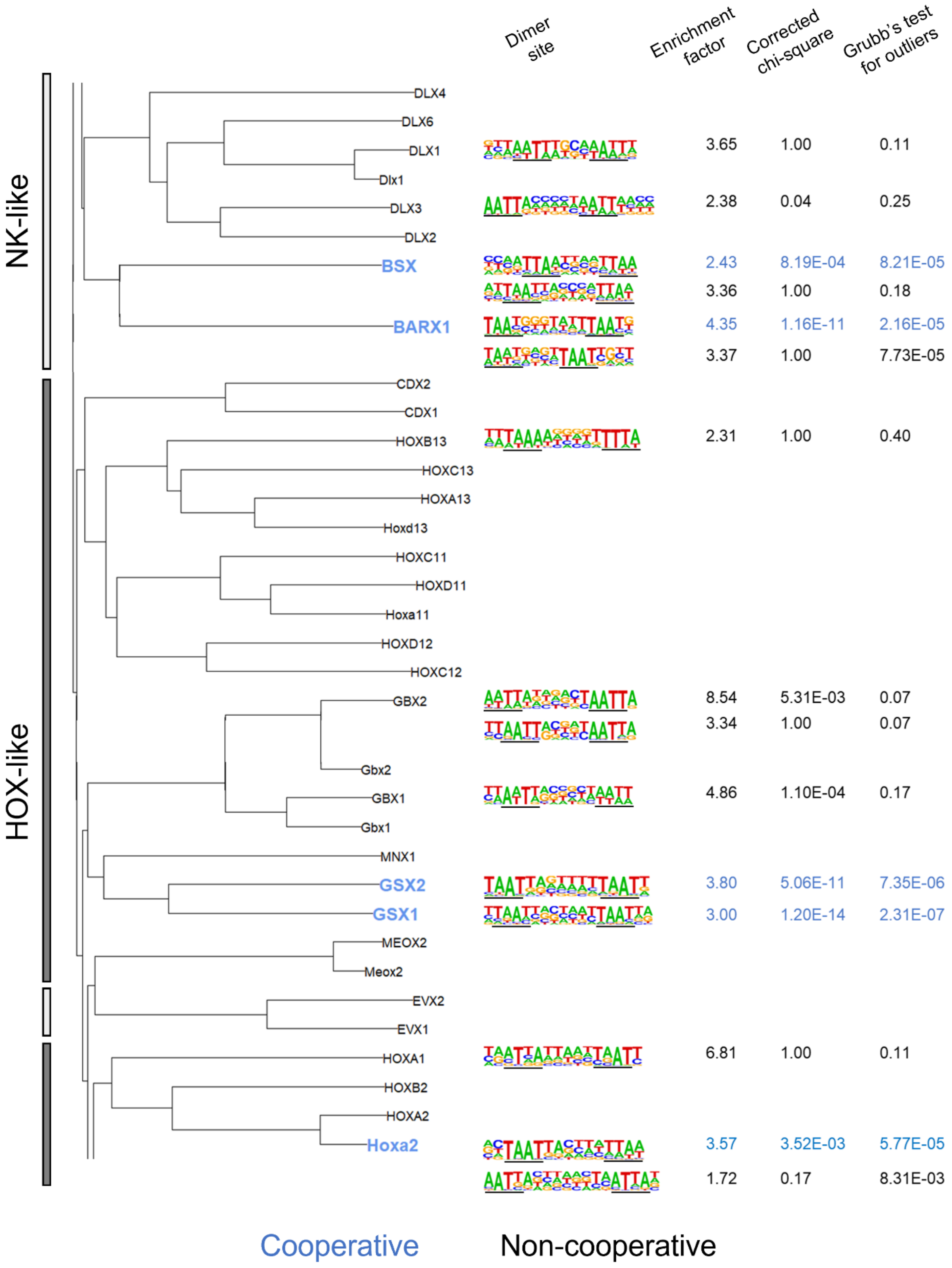

Dimer site
Enrichment factor
Corrected chi-square
Grubb's test for outliers
NK-like
HOX-like
DLX4
DLX6
DLX1
Dlx1
DLX3
DLX2
BSX
BARX1
CDX2
CDX1
HOXB13
HOXC13
HOXA13
Hoxd13
HOXC11
HOXD11
Hoxa11
HOXD12
HOXC12
GBX2
Gbx2
GBX1
Gbx1
MNX1
GSX2
GSX1
MEOX2
Meox2
EVX2
EVX1
HOXA1
HOXB2
HOXA2
Hoxa2
3.65 1.00 0.11
2.38 0.04 0.25
2.43 8.19E-04 8.21E-05
3.36 1.00 0.18
4.35 1.16E-11 2.16E-05
3.37 1.00 7.73E-05
2.31 1.00 0.40
8.54 5.31E-03 0.07
3.34 1.00 0.07
4.86 1.10E-04 0.17
3.80 5.06E-11 7.35E-06
3.00 1.20E-14 2.31E-07
6.81 1.00 0.11
3.57 3.52E-03 5.77E-05
1.72 0.17 8.31E-03
Cooperative
Non-cooperative

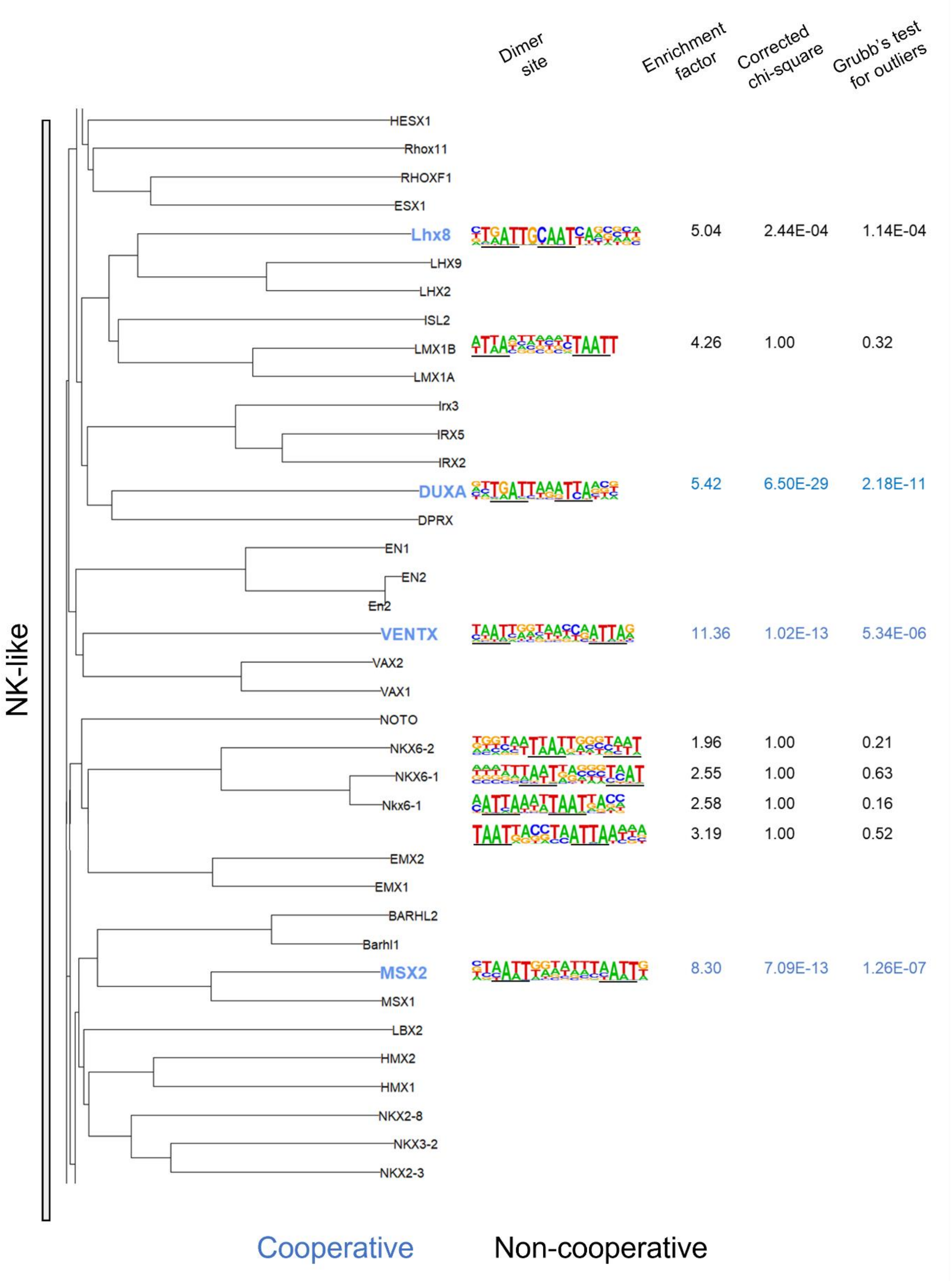
Dimer site
Enrichment factor
Corrected chi-square
Grubb's test for outliers
NK-like
HESX1
Rhox11
RHOXF1
ESX1
Lhx8
5.04
2.44E-04
1.14E-04
LHX9
LHX2
ISL2
LMX1B
4.26
1.00
0.32
LMX1A
Irx3
IRX5
IRX2
DUXA
5.42
6.50E-29
2.18E-11
DPRX
EN1
EN2
En2
VENTX
11.36
1.02E-13
5.34E-06
VAX2
VAX1
NOTO
NKX6-2
1.96
1.00
0.21
NKX6-1
2.55
1.00
0.63
Nkx6-1
2.58
1.00
0.16
3.19
1.00
0.52
EMX2
EMX1
BARHL2
Barhl1
MSX2
8.30
7.09E-13
1.26E-07
MSX1
LBX2
HMX2
HMX1
NKX2-8
NKX3-2
NKX2-3
Cooperative
Non-cooperative

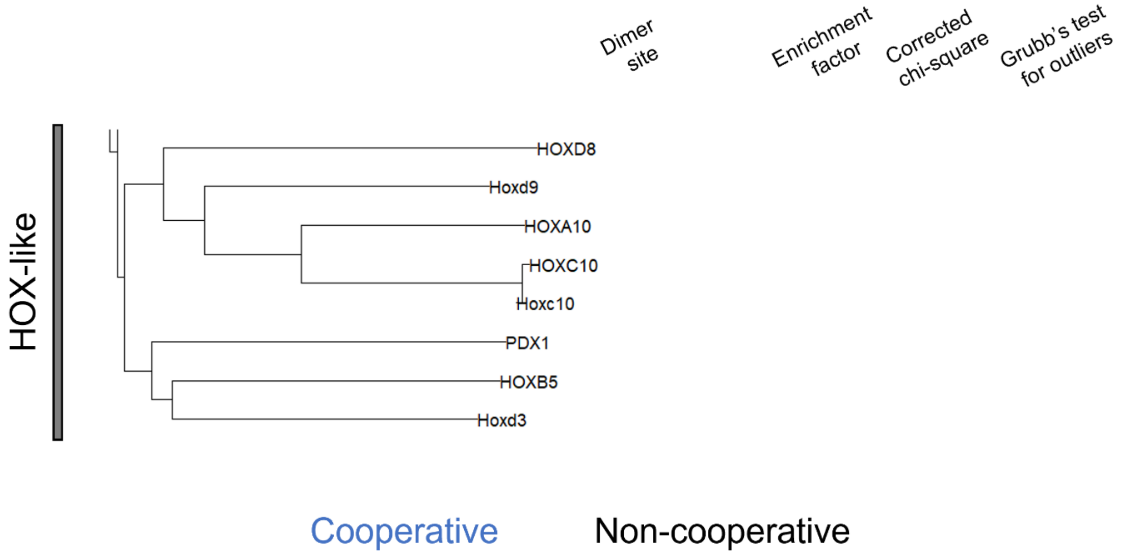

Supplementary Figure 3. **The human and mouse homeodomain proteins are predicted to bind cooperatively to several unique binding site arrangements.** Summary of the Cooperativity Predictor results of the HOX-like, NK-like, and Paired-like human (88 in total) and mouse (24 in total) TFs analyzed. Those TFs that were predicted to bind cooperatively are listed in blue, bold text. All identified dimer motifs are listed with the top 4mers for each identified dimer site underlined. All enrichment factors, chi-square test for independence p-values, and Grubb's test for outlier p-values are shown. Values are shown in blue if the dimer site passed all cooperativity criteria.

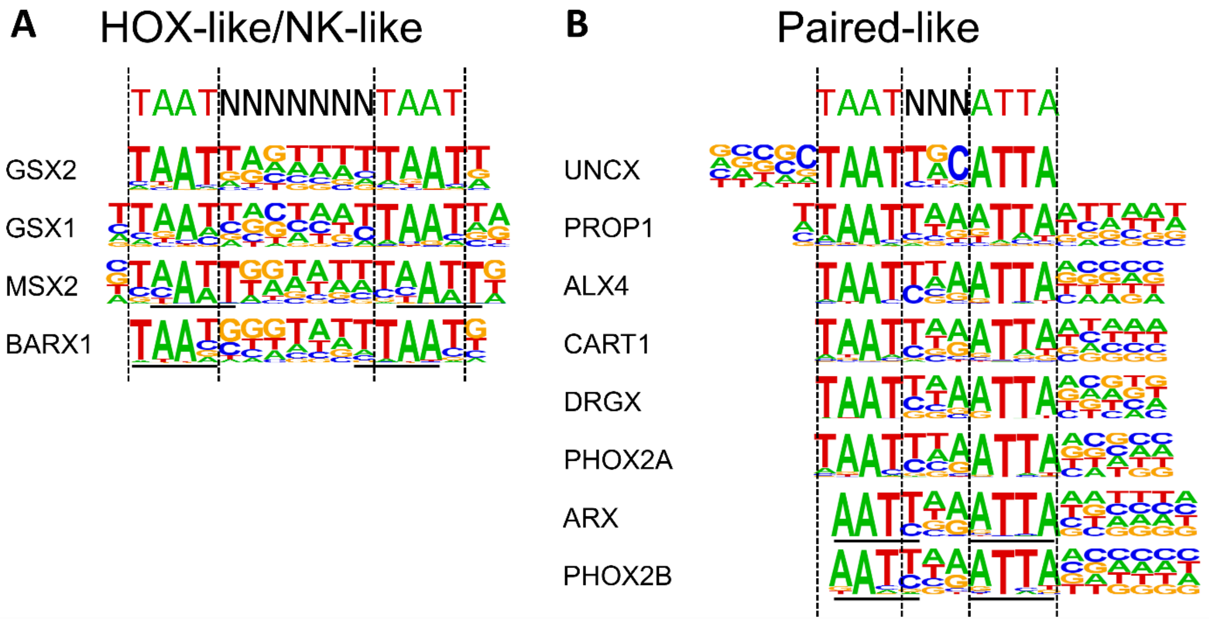

Supplementary Figure 4. Motif alignment reveals a high-degree of similarity between the GSX2, GSX1, MSX2 and BARX1 motifs as well as the Paired-like motifs. (A) Select members of the HOX-like and NK-like classes bound to TAATs separated by a 7bp spacer. Due to slight variation in information content, MSX2 had AATTs called as top 4mers and BARX1 had a TAAT and TTAA called as top 4mers separated by 6 bps. **(B)** Motif alignment of the cooperative Paired-like factors reveals a highly similar TAATNNNATTA motif, even for those with a defined Homer motif starting 1bp after the start of the Paired-like motif in the case of ARX and PHOX2B. In these cases, the Cooperativity Predictor defined the ARX and PHOX2B motifs as a 2bp spacer rather than the typical 3bp spacer. However, all these motifs are largely the same and can be grouped and classified as a single unique binding arrangement.

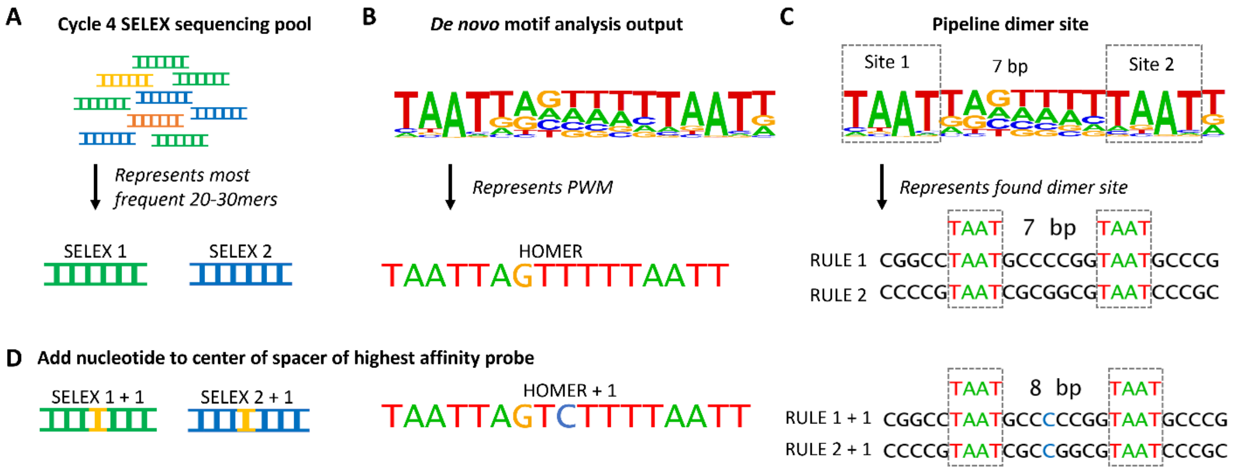

Supplementary Figure 5. Schematics of probe design for TF cooperativity tests. Five EMSA probes were designed for each TF: **(A)** The SELEX 1 and SELEX 2 probes represented the two most frequent randomers from the cycle 4 sequencing pool. **(B)** The HOMER probe represents the optimal sequence based upon the sequence information content from the PWM identified by Homer. **(C)** The rule probes correspond to the consensus sequence found by Cooperativity Predictor in which the two 4mers are distanced and flanked with GC sequence. **(D)** A single bp was then added to the center of the spacer of the probe that had the most binding in the initial screen.

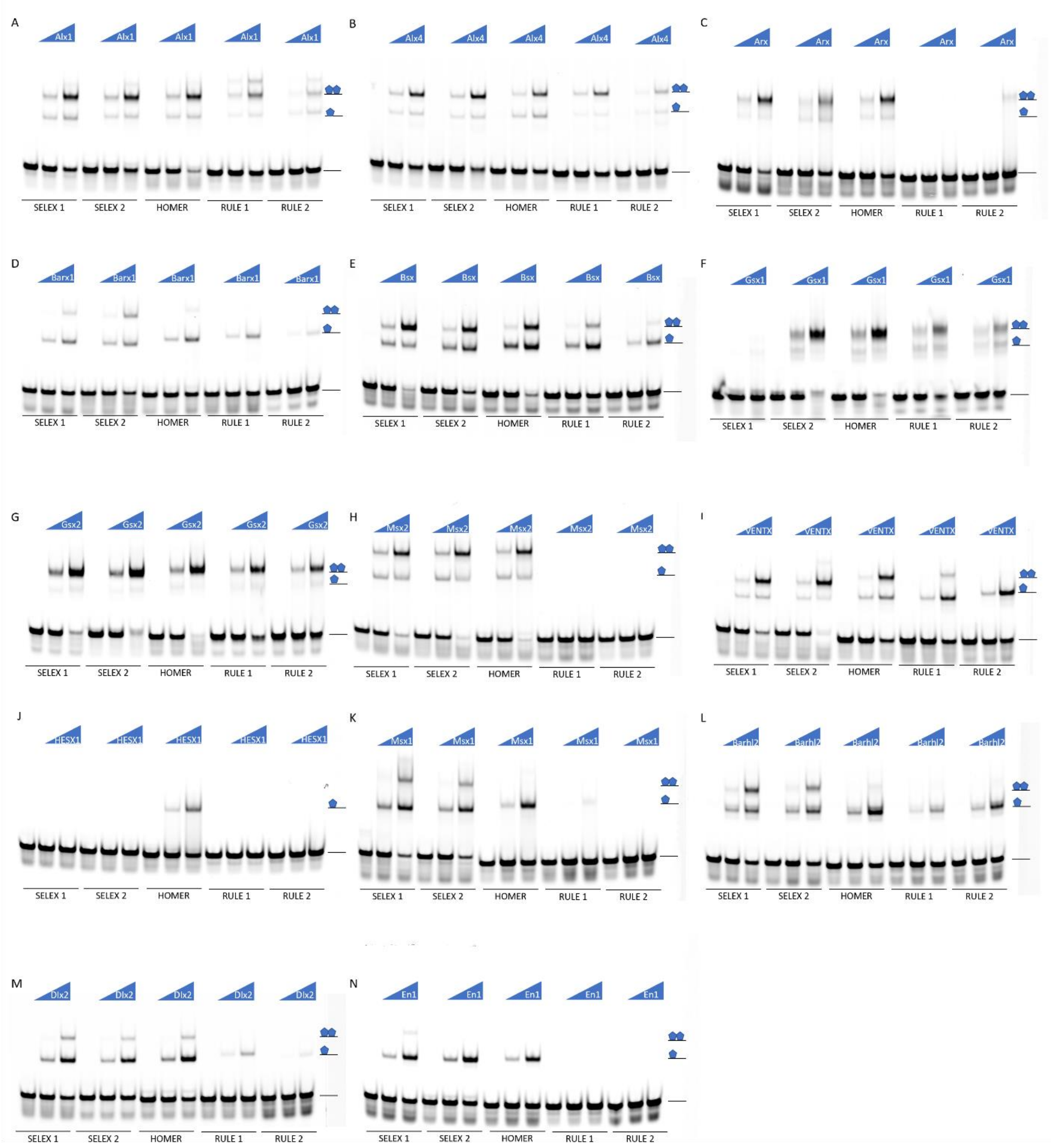

Supplementary Figure 6. **DNA binding assays of 14 HD TFs tested on 5 probes containing the predicted dimer sites.** The 9 TFs tested in **(A-I)** were predicted to bind the identified dimer site in a cooperative manner, whereas the 5 TFs tested in **(J-N)** were not predicted to bind cooperatively to the identified dimer site. Each binding reaction used 34 nM of the indicated labeled DNA probe and probe sequences are listed in Supplementary Table 7. Two concentrations were tested for each protein using a four-fold step between the low and high lane as noted below. Schematics of the predicted protein-DNA complexes (unbound DNA, monomer complex, and dimer complex) are shown to the right of each gel. **(A)**

Alx1 probes were tested using 0, 62.5, and 250 nM of Alx1 (105-220) (the mouse ortholog of CART1) protein. The Alx1 SELEX 2 probe was chosen for the Tau cooperativity analysis. **(B)** Alx4 probes were tested using 0, 12.5 and 50 nM of Alx4 (128-291). The Alx4 SELEX 1 probe was chosen for the Tau cooperativity analysis. **(C)** Arx probes were tested using 0, 150, and 600 nM of Arx (292-420) protein. The Arx SELEX 1 probe was chosen for Tau cooperativity analysis. **(D)** Barx1 probes were tested using 0, 250, and 1000 nM of Barx1 (95-221) protein. The Barx1 SELEX 2 probe was chosen for Tau cooperativity analysis. **(E)** Bsx probes were tested using 0, 150, and 600 nM of Bsx (84-193) protein. The Bsx SELEX 1 probe was chosen for Tau cooperativity analysis. **(F)** Gsx1 probes were tested using 0, 150, and 600 nM of Gsx1 (127-261) protein. The Gsx1 HOMER probe was chosen for Tau cooperativity analysis. **(G)** Gsx2 probes were tested using 0, 100, and 400 nM of Gsx2 (167-305) protein. The Gsx2 HOMER probe was chosen for Tau cooperativity analysis. **(H)** Msx2 probes were tested using 0, 455, and 1820 nM of Msx2 (99-227) protein. The Msx2 SELEX 2 probe was chosen for Tau cooperativity analysis. **(I)** VENTX probes were tested using 0, 25, and 100 nM of VENTX (69-178) protein. The VENTX SELEX 1 probe was chosen for Tau cooperativity analysis. **(J)** HESX1 probes were tested using 0, 325, and 1300 nM of HESX1 (65-185) protein. HESX1 only successfully bound to the HOMER probe, and this probe was selected for Tau cooperativity analysis. **(K)** MSX1 probes were tested using 0, 455, and 1820 nM of MSX1 (131-258) protein. The MSX1 SELEX 1 probe was chosen for Tau cooperativity analysis. **(L)** Barhl2 probes were tested using 0, 150, and 600 nM of Barhl2 (190-316) protein. The Barhl2 SELEX 1 probe was chosen for Tau cooperativity analysis. **(M)** Dlx2 probes were tested using 0, 20, and 80 nM of Dlx2 (137-229) protein. The Dlx2 SELEX 1 probe was chosen for Tau cooperativity analysis. **(N)** En1 probes were tested using 0, 150, and 600 nM of En1 (290-400) protein. The En1 SELEX probe was chosen for Tau cooperativity testing.

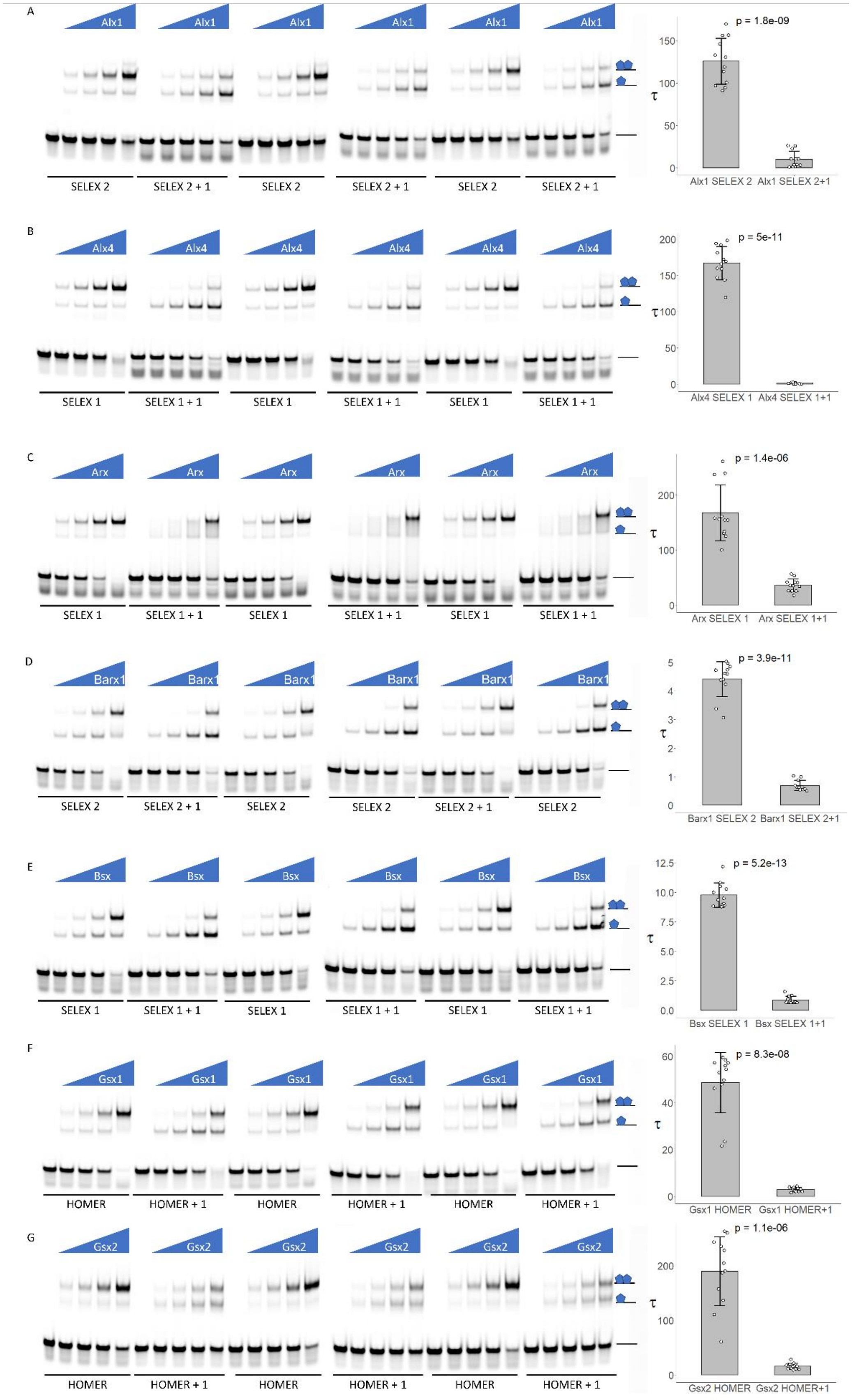
A
Alx1
SELEX 2
SELEX 2 + 1
p = 1.8e-09
τ
Alx1 SELEX 2
Alx1 SELEX 2+1
B
Alx4
SELEX 1
SELEX 1 + 1
p = 5e-11
Alx4 SELEX 1
Alx4 SELEX 1+1
C
Arx
p = 1.4e-06
Arx SELEX 1
Arx SELEX 1+1
D
Barx1
p = 3.9e-11
Barx1 SELEX 2
Barx1 SELEX 2+1
E
Bsx
p = 5.2e-13
Bsx SELEX 1
Bsx SELEX 1+1
F
Gsx1
HOMER
HOMER + 1
p = 8.3e-08
Gsx1 HOMER
Gsx1 HOMER+1
G
Gsx2
p = 1.1e-06
Gsx2 HOMER
Gsx2 HOMER+1

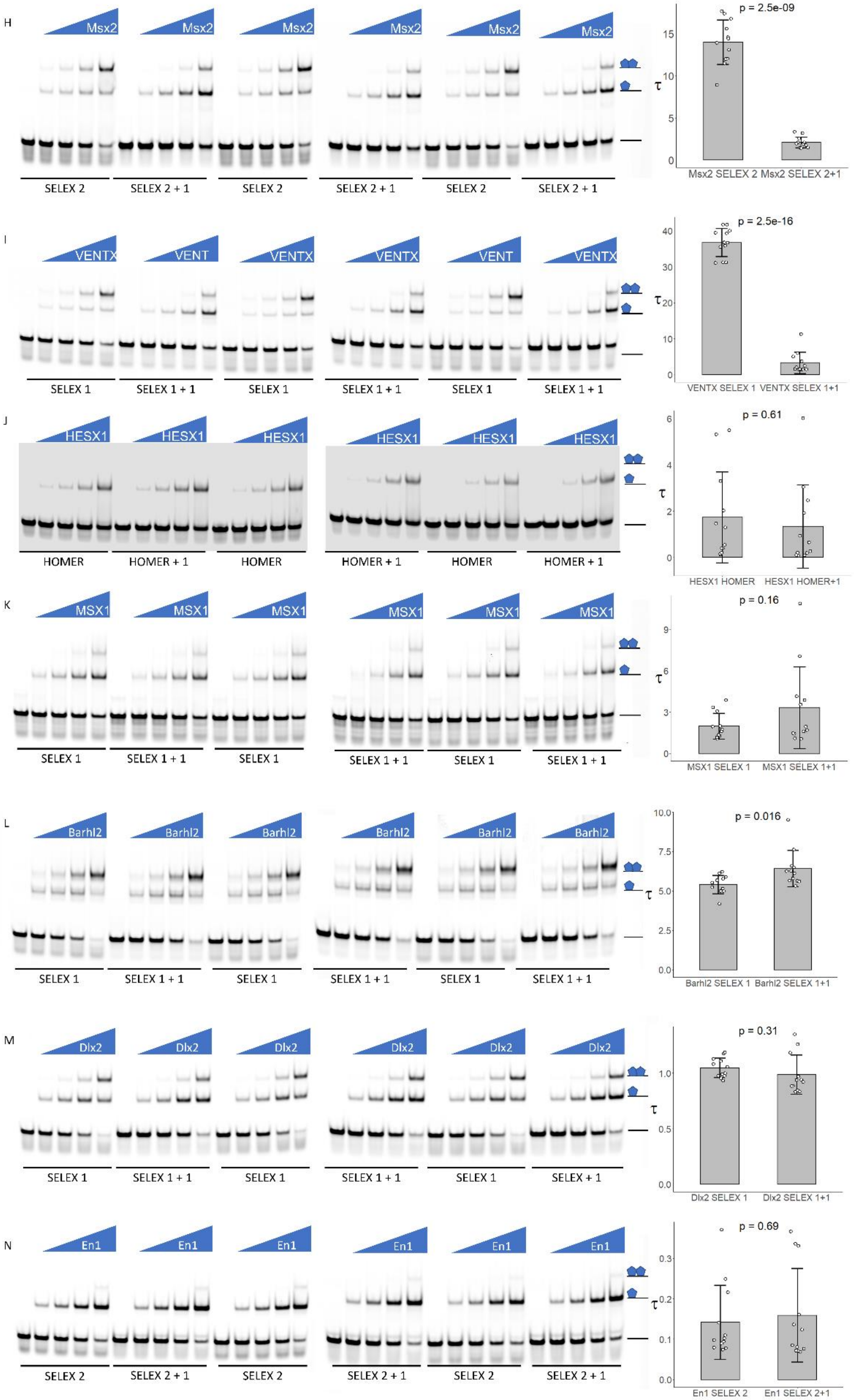
H
Msx2
SELEX 2
SELEX 2 + 1
p = 2.5e-09
τ
Msx2 SELEX 2
Msx2 SELEX 2+1
I
VENTX
VENT
SELEX 1
SELEX 1 + 1
p = 2.5e-16
VENTX SELEX 1
VENTX SELEX 1+1
J
HESX1
HOMER
HOMER + 1
p = 0.61
HESX1 HOMER
HESX1 HOMER+1
K
MSX1
SELEX 1
SELEX 1 + 1
p = 0.16
MSX1 SELEX 1
MSX1 SELEX 1+1
L
Barhl2
SELEX 1
SELEX 1 + 1
p = 0.016
Barhl2 SELEX 1
Barhl2 SELEX 1+1
M
Dlx2
SELEX 1
SELEX 1 + 1
SELEX + 1
p = 0.31
Dlx2 SELEX 1
Dlx2 SELEX 1+1
N
En1
SELEX 2
SELEX 2 + 1
p = 0.69
En1 SELEX 2
En1 SELEX 2+1

Supplementary Figure 7. HD TFs predicted to bind DNA cooperatively bound cooperatively in a spacer specific manner. Each EMSA has 30 total lanes in which the predicted dimer probe and the probe with the added nucleotide in between the individual sites (the +1 probe) were tested at 4 concentrations in triplicate. 34 nM of fluorescent probe was used in each lane. The sequences of the DNA probes used are listed in Supplementary Table 7. We aimed to saturate 80-95% of the probe with the highest TF concentration lane and diluted the protein 2-fold for each preceding lane. However, the same TF concentrations were used on the dimer and +1 probes for each protein tested. Schematics of the DNA complexes (unbound, monomer bound, and dimer bound) are shown to the right of the gel. All calculated Tau cooperativity factors are summarized in Figure 3B-C and Supplementary Table 8. Bar graphs depict the averaged Tau cooperativity factor for each TF with each dot representing a Tau factor from an individual binding reaction (n = 12 for each group). Error bars denote standard deviation. Tau cooperativity factors were compared with two-sided unpaired student t-tests. **(A-I)** The Tau cooperativity factor of each significantly decreased after the addition of a single nucleotide to the spacer for all the predicted cooperative TFs, Alx1-VENTX. **(J-K; M-N)** In contrast, the Tau factor either did not significantly change for the non-cooperative TFs: HESX1, MSX1, Dlx2, and En1, or **(L)** increased slightly in the case of Barhl2. The Tau cooperativity values were calculated using the following protein concentrations for each protein: **(A)** Alx1 (amino acids 105-220) = 0, 31.25, 62.5, 125, and 250 nM. **(B)** Alx4 (amino acids 128-291) = 0, 12.5, 25, 50, and 100 nM. **(C)** Arx (amino acids 292-420) = 0, 31.25, 62.5, 125, and 250 nM. **(D)** Barx1 (amino acids 95-221) = 0, 20, 40, 80, and 160 nM. **(E)** Bsx (amino acids 84-193) = 0, 12.5, 25, 50, and 100 nM. **(F)** Gsx1 (amino acids 127-261) = 0, 50, 100, 200, and 400 nM. **(G)** Gsx2 (amino acids 167-305) = 0, 31.25, 62.5, 125, and 250 nM. **(H)** Msx2 (amino acids 99-227) = 0, 12.5, 25, 50, and 100 nM. **(I)** VENTX (amino acids 69-178) = 0, 12.5, 25, 50, and 100 nM. **(J)** HESX1 (amino acids 65-185) = 0, 132.5, 325, 650, and 1300 nM. **(K)** MSX1 (amino acids 131-258) = 0, 227.5, 455, 910, and 1820 nM. **(L)** Barhl2 (amino acids 190-316) = 0, 200, 400, 800, and 1600 nM. **(M)** Dlx2 (amino acids 137-229) = 0, 20, 40, 80, and 160 nM. **(N)** En1 (amino acids 290-400) = 0, 156.25, 312.5, 625, 1250 nM.

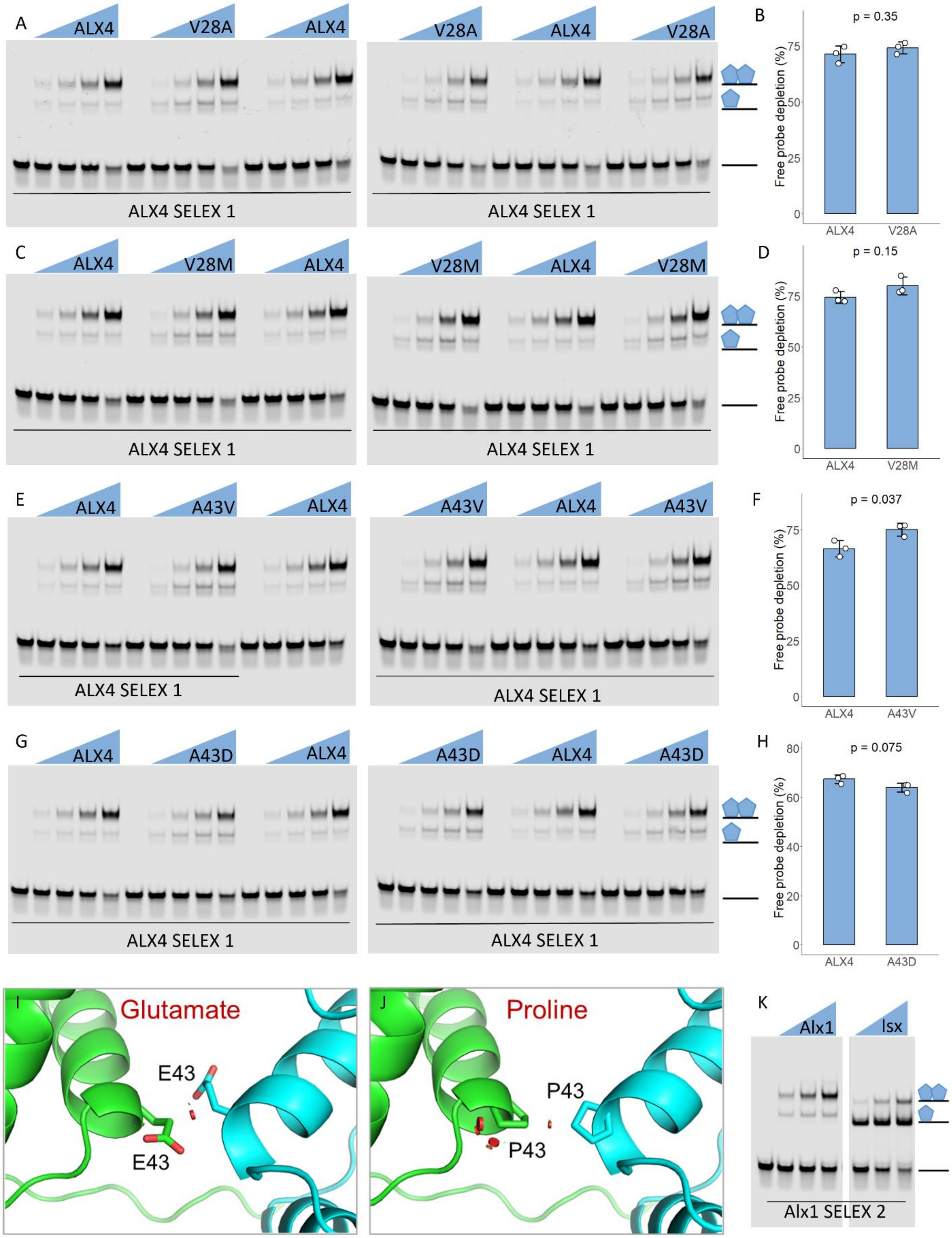

Supplementary Figure 8. The 28th and 43rd residues in the HD influence the magnitude of cooperativity but do not fully explain the differential cooperative behavior between the Paired-like

TFs. (A-H) Four ALX4 variants in which either position 28 or 43 was changed to a residue found in a non-cooperative TF were tested in EMSA. 34 nM of probe was used in each lane. The DNA sequence of the probe is listed in Supplementary Table 7. We aimed to bind 80-95% of the probe with the highest TF concentration lane and diluted the protein 2-fold for each preceding lane. Schematics of the DNA complexes (unbound, monomer bound, and dimer bound) are shown to the right of the gel. Experiments were performed in triplicate with the following concentrations: ALX4 (amino acids 169-303) = 0, 18.75, 37.5, 75, and 150 nM. The cooperativity variants tested influenced cooperativity of the protein but had very little impact on DNA binding affinity as evidenced by free probe depletion. The percent free probe depleted at the 150nM concentration for each experiment was quantified and compared with an unpaired, two-sided student t-test. **(I-J)** A glutamic acid and proline in the 43rd position of the HD as in GSC2 and PITX1 respectively would cause considerable clashes between residues as shown by the red discs. These clashes are predicted to reduce cooperativity similar to valine and aspartic acid at these positions. **(K)** Isx does not bind cooperatively to a Paired-like site despite having the key residues at positions 28 and 43 required for cooperativity. Alx1 (amino acids 105-220) = 0, 62.5, 125, and 250 nM. Isx (amino acids 54-163) = 0, 62.5, 125, and 250 nM.

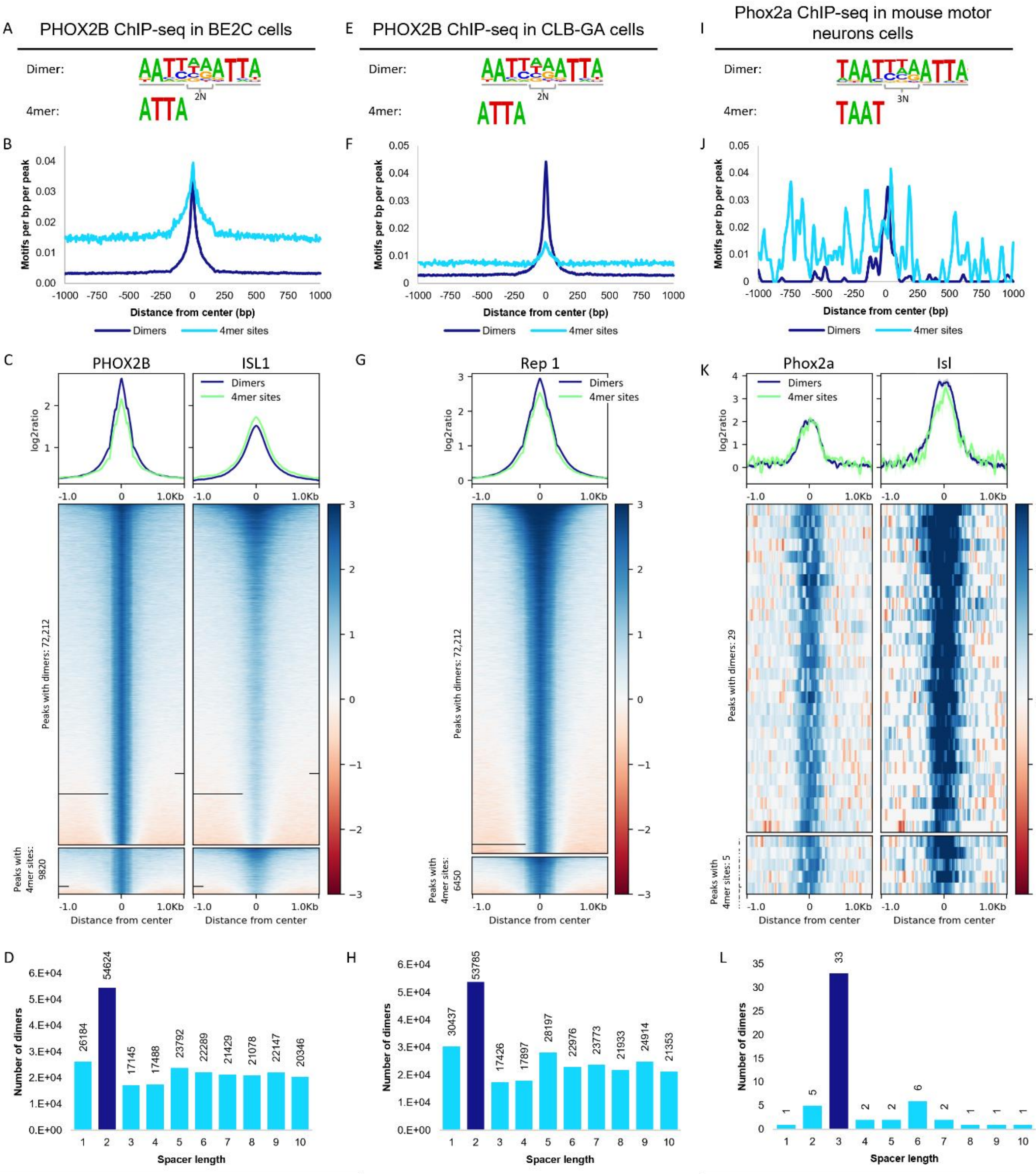

Supplementary Figure 9. PHOX factors bind to dimer sites in genomic binding assays. (A-H) Analysis of PHOX2B ChIP-seq data in two neuroblastoma cell lines revealed similar findings to the ChIP-seq performed in KELLY cells (Figure 5). **(A-B,E-F)** PHOX2B had high motif enrichment for both dimer and monomer sites in respective peaks. **(C,G)** Peaks with dimer sites had higher PHOX2B binding signal than peaks with only 4mer sites. This was not true of the ISL1 binding signal in BE2C cells, suggesting that this dimerization is a result of PHOX2B homodimers rather than PHOX2B and ISL1 heterodimers. **(D,H)** Dimer sites consisting of 4mers 2bps apart were highly enriched as predicted based on the HT-SELEX data. **(I-J)**

High doxycycline induction of both Phox2a and its heterodimerization partner, Isl1, made interpretation of Phox2a ChIP-seq in this context challenging as the homodimerization of Phox2a was most likely masked by Phox2a and Isl heterodimerization. **(I-J)** Phox2a had high enrichment of the dimer site at the peak centers whereas monomer sites were not enriched at only the peak centers. Note, there were very few peaks called for the Phox2a dataset and only five peaks contained a 4mer but not a dimer site. **(K)** The peaks with dimer sites did not have a higher binding signal in this dataset, but this may be attributed to low peak number. **(L)** There was high enrichment of dimer sites at a 3bp spacer which is consistent with the heterodimer site previously detected (1).

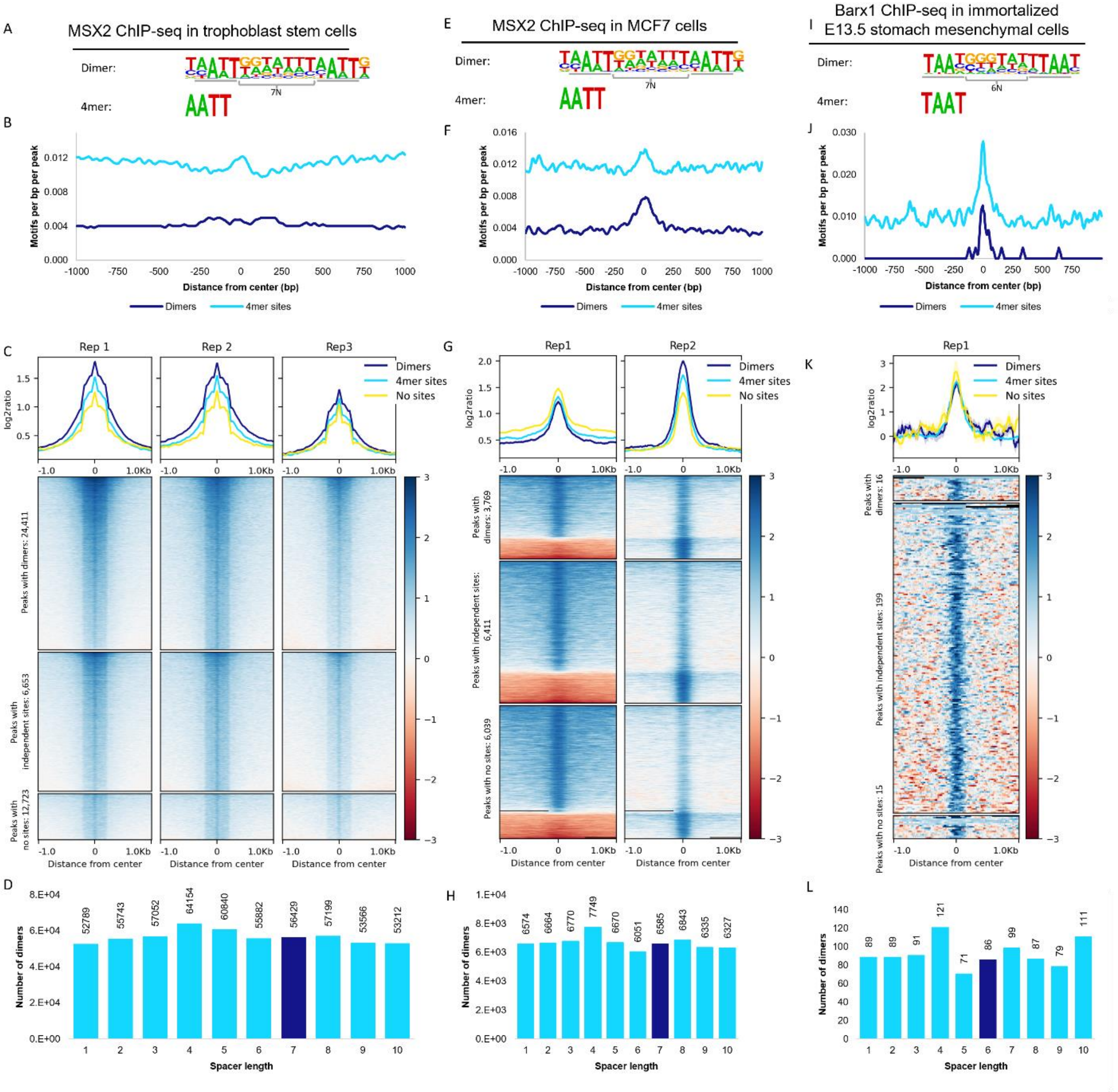

Supplementary Figure 10. MSX2 and Barx1 genomic binding assays did not provide evidence of cooperative binding *in vivo*. (A-B,E-F) Neither the MSX2 ChIP-seq datasets in human trophoblast stem cells nor MCF7 cells showed high enrichment of the dimer or 4mer site. This made comparison of binding signal **(C,G)** and the COSMO analysis **(D,H)** difficult to interpret. As such, little evidence of MSX2 binding to cooperative sites was found. **(I-J)** FLAG-Barx1 ChIP-seq in immortalized E13.5 stomach mesenchymal cells revealed high enrichment of both dimer and 4mer sites. **(K)** There were few peaks called for this dataset and very few of these peaks contained dimer sites. Surprisingly, peaks without either site had the highest binding signal, and **(L)** dimer sites with a 6 bp spacer were not enriched in the COSMO analysis.

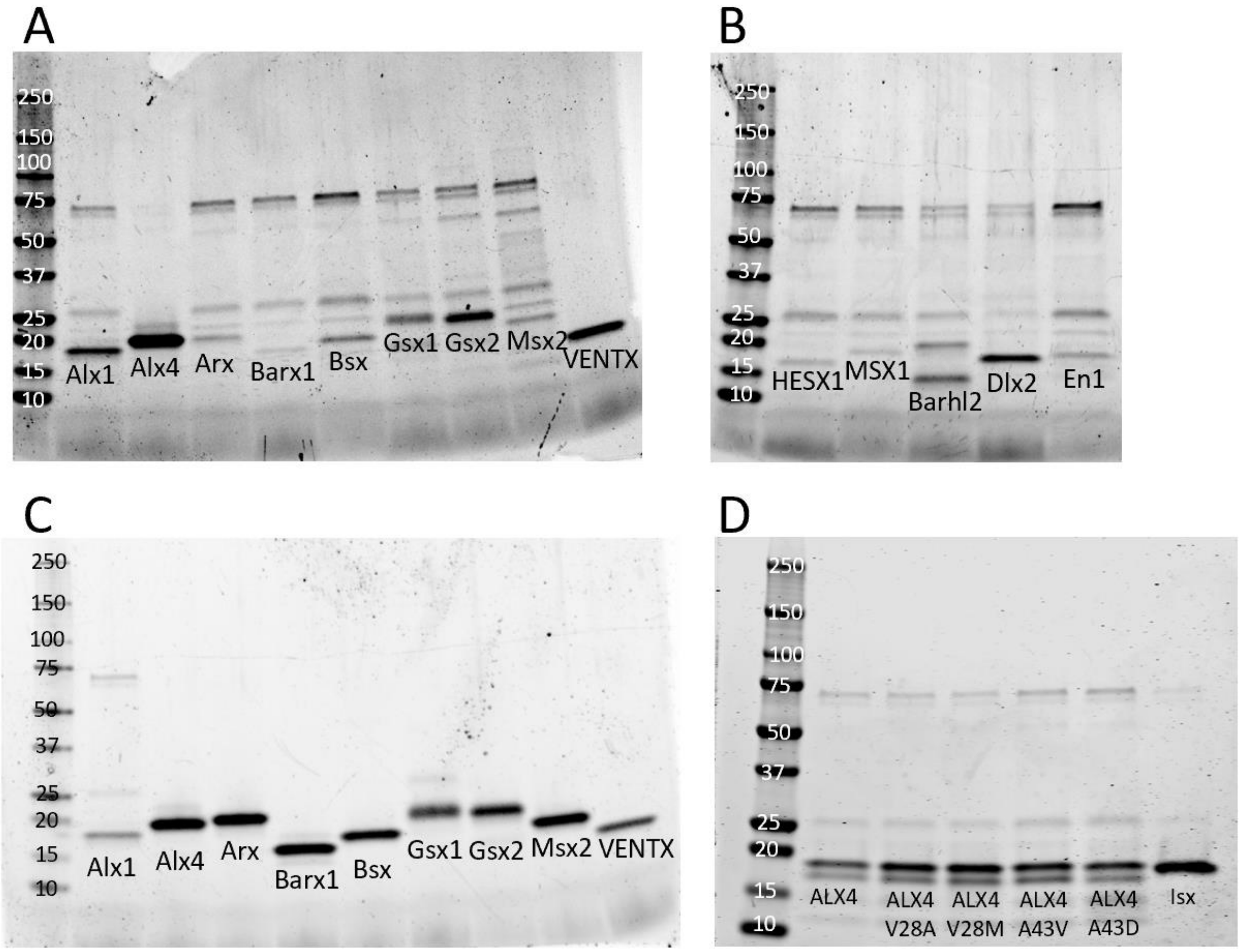

Supplementary Figure 11. SDS PAGE gels of proteins stained with GelCode Blue stain reagent (Thermo Scientific) shows purity of proteins used in EMSAs. (A) Proteins used in generating the EMSAs in Supplementary Figure 6. **(B)** Proteins used in generating the EMSAs in Supplementary Figure 6 and Supplementary Figure 7. **(C)** Proteins used in generating the EMSAs in Supplementary Figure 7. Several of the proteins from (A) were repurified between the generation of and Supplementary Figure 7. None of the proteins from (B) were repurified. **(D)** Proteins used in generating the EMSAs in Supplementary Figure 8.

SUPPLEMENTARY INFORMATION

> pET14P

TTCTTGAAGACGAAAGGGCCTCGTGATACGCCTATTTTTATAGGTTAATGTCATGATAATAATGGTTTCTTAGACGT
CAGGTGGCACTTTTCGGGGAAATGTGCGCGGAACCCCTATTTGTTTATTTTTCTAAATACATTCAAATATGTATCCG
CTCATGAGACAATAACCCTGATAAATGCTTCAATAATATTGAAAAAGGAAGAGTATGAGTATTCAACATTTCCGTGT
CGCCCTTATTCCCTTTTTTGCGGCATTTTGCCTTCCTGTTTTTGCTCACCCAGAAACGCTGGTGAAAGTAAAAGATG
CTGAAGATCAGTTGGGTGCACGAGTGGGTTACATCGAACTGGATCTCAACAGCGGTAAGATCCTTGAGAGTTTTCGC
CCCGAAGAACGTTTTCCAATGATGAGCACTTTTAAAGTTCTGCTATGTGGCGCGGTATTATCCCGTGTTGACGCCGG
GCAAGAGCAACTCGGTCGCCGCATACACTATTCTCAGAATGACTTGGTTGAGTACTCACCAGTCACAGAAAAGCATC
TTACGGATGGCATGACAGTAAGAGAATTATGCAGTGCTGCCATAACCATGAGTGATAACACTGCGGCCAACTTACTT
CTGACAACGATCGGAGGACCGAAGGAGCTAACCGCTTTTTTGCACAACATGGGGGATCATGTAACTCGCCTTGATCG
TTGGGAACCGGAGCTGAATGAAGCCATACCAAACGACGAGCGTGACACCACGATGCCTGCAGCAATGGCAACAACGT
TGCGCAAACTATTAACTGGCGAACTACTTACTCTAGCTTCCCGGCAACAATTAATAGACTGGATGGAGGCGGATAAA
GTTGCAGGACCACTTCTGCGCTCGGCCCTTCCGGCTGGCTGGTTTATTGCTGATAAATCTGGAGCCGGTGAGCGTGG
GTCTCGCGGTATCATTGCAGCACTGGGGCCAGATGGTAAGCCCTCCCGTATCGTAGTTATCTACACGACGGGGAGTC
AGGCAACTATGGATGAACGAAATAGACAGATCGCTGAGATAGGTGCCTCACTGATTAAGCATTGGTAACTGTCAGAC
CAAGTTTACTCATATATACTTTAGATTGATTTAAAACTTCATTTTTAATTTAAAAGGATCTAGGTGAAGATCCTTTT
TGATAATCTCATGACCAAAATCCCTTAACGTGAGTTTTCGTTCCACTGAGCGTCAGACCCCGTAGAAAAGATCAAAG
GATCTTCTTGAGATCCTTTTTTTCTGCGCGTAATCTGCTGCTTGCAAACAAAAAAACCACCGCTACCAGCGGTGGTT
TGTTTGCCGGATCAAGAGCTACCAACTCTTTTTCCGAAGGTAACTGGCTTCAGCAGAGCGCAGATACCAAATACTGT
CCTTCTAGTGTAGCCGTAGTTAGGCCACCACTTCAAGAACTCTGTAGCACCGCCTACATACCTCGCTCTGCTAATCC
TGTTACCAGTGGCTGCTGCCAGTGGCGATAAGTCGTGTCTTACCGGGTTGGACTCAAGACGATAGTTACCGGATAAG
GCGCAGCGGTCGGGCTGAACGGGGGGTTCGTGCACACAGCCCAGCTTGGAGCGAACGACCTACACCGAACTGAGATA
CCTACAGCGTGAGCTATGAGAAAGCGCCACGCTTCCCGAAGGGAGAAAGGCGGACAGGTATCCGGTAAGCGGCAGGG
TCGGAACAGGAGAGCGCACGAGGGAGCTTCCAGGGGGAAACGCCTGGTATCTTTATAGTCCTGTCGGGTTTCGCCAC
CTCTGACTTGAGCGTCGATTTTTGTGATGCTCGTCAGGGGGGCGGAGCCTATGGAAAAACGCCAGCAACGCGGCCTT
TTTACGGTTCCTGGCCTTTTGCTGGCCTTTTGCTCACATGTTCTTTCCTGCGTTATCCCCTGATTCTGTGGATAACC
GTATTACCGCCTTTGAGTGAGCTGATACCGCTCGCCGCAGCCGAACGACCGAGCGCAGCGAGTCAGTGAGCGAGGAA
GCGGAAGAGCGCCTGATGCGGTATTTTCTCCTTACGCATCTGTGCGGTATTTCACACCGCATATATGGTGCACTCTC
AGTACAATCTGCTCTGATGCCGCATAGTTAAGCCAGTATACACTCCGCTATCGCTACGTGACTGGGTCATGGCTGCG
CCCCGACACCCGCCAACACCCGCTGACGCGCCCTGACGGGCTTGTCTGCTCCCGGCATCCGCTTACAGACAAGCTGT
GACCGTCTCCGGGAGCTGCATGTGTCAGAGGTTTTCACCGTCATCACCGAAACGCGCGAGGCAGCTGCGGTAAAGCT
CATCAGCGTGGTCGTGAAGCGATTCACAGATGTCTGCCTGTTCATCCGCGTCCAGCTCGTTGAGTTTCTCCAGAAGC

GTTAATGTCTGGCTTCTGATAAAGCGGGCCATGTTAAGGGCGGTTTTTTCCTGTTTGGTCACTGATGCCTCCGTGTA
AGGGGGATTTCTGTTCATGGGGGTAATGATACCGATGAAACGAGAGAGGATGCTCACGATACGGGTTACTGATGATG
AACATGCCCGGTTACTGGAACGTTGTGAGGGTAAACAACTGGCGGTATGGATGCGGCGGGACCAGAGAAAAATCACT
CAGGGTCAATGCCAGCGCTTCGTTAATACAGATGTAGGTGTTCCACAGGGTAGCCAGCAGCATCCTGCGATGCAGAT
CCGGAACATAATGGTGCAGGGCGCTGACTTCCGCGTTTCCAGACTTTACGAAACACGGAAACCGAAGACCATTCATG
TTGTTGCTCAGGTCGCAGACGTTTTGCAGCAGCAGTCGCTTCACGTTCGCTCGCGTATCGGTGATTCATTCTGCTAA
CCAGTAAGGCAACCCCGCCAGCCTAGCCGGGTCCTCAACGACAGGAGCACGATCATGCGCACCCGTGGCCAGGACCC
AACGCTGCCCGAGATGCGCCGCGTGCGGCTGCTGGAGATGGCGGACGCGATGGATATGTTCTGCCAAGGGTTGGTTT
GCGCATTCACAGTTCTCCGCAAGAATTGATTGGCTCCAATTCTTGGAGTGGTGAATCCGTTAGCGAGGTGCCGCCGG
CTTCCATTCAGGTCGAGGTGGCCCGGCTCCATGCACCGCGACGCAACGCGGGGAGGCAGACAAGGTATAGGGCGGCG
CCTACAATCCATGCCAACCCGTTCCATGTGCTCGCCGAGGCGGCATAAATCGCCGTGACGATCAGCGGTCCAGTGAT
CGAAGTTAGGCTGGTAAGAGCCGCGAGCGATCCTTGAAGCTGTCCCTGATGGTCGTCATCTACCTGCCTGGACAGCA
TGGCCTGCAACGCGGGCATCCCGATGCCGCCGGAAGCGAGAAGAATCATAATGGGGAAGGCCATCCAGCCTCGCGTC
GCGAACGCCAGCAAGACGTAGCCCAGCGCGTCGGCCGCCATGCCGGCGATAATGGCCTGCTTCTCGCCGAAACGTTT
GGTGGCGGGACCAGTGACGAAGGCTTGAGCGAGGGCGTGCAAGATTCCGAATACCGCAAGCGACAGGCCGATCATCG
TCGCGCTCCAGCGAAAGCGGTCCTCGCCGAAAATGACCCAGAGCGCTGCCGGCACCTGTCCTACGAGTTGCATGATA
AAGAAGACAGTCATAAGTGCGGCGACGATAGTCATGCCCCGCGCCCACCGGAAGGAGCTGACTGGGTTGAAGGCTCT
CAAGGGCATCGGTCGACGCTCTCCCTTATGCGACTCCTGCATTAGGAAGCAGCCCAGTAGTAGGTTGAGGCCGTTGA
GCACCGCCGCCGCAAGGAATGGTGCATGCAAGGAGATGGCGCCCAACAGTCCCCCGGCCACGGGGCCTGCCACCATA
CCCACGCCGAAACAAGCGCTCATGAGCCCGAAGTGGCGAGCCCGATCTTCCCCATCGGTGATGTCGGCGATATAGGC
GCCAGCAACCGCACCTGTGGCGCCGGTGATGCCGGCCACGATGCGTCCGGCGTAGAGGATCGAGATCTCGATCCCGC
GAAATTAATACGACTCACTATAGGGAGACCACAACGGTTTCCCTCTAGAAATAATTTTGTTTAACTTTAAGAAGGAG
ATATACCATGGGCAGCAGCCATCATCATCATCATCACAGCAGCGGCCTGGAAGTTCTGTTCCAGGGGCCCGCGGCCG
CACATATGGGATCCCTCGAGTAAGCTGCTAACAAAGCCCGAAAGGAAGCTGAGTTGGCTGCTGCCACCGCTGAGCAA
TAACTAGCATAACCCCTTGGGGCCTCTAAACGGGTCTTGAGGGGTTTTTTGCTGAAAGGAGGAACTATATCCGGATA
TCCACAGGACGGGTGTGGTCGCCATGATCGCGTAGTCGATAGTGGCTCCAAGTAGCGAAGCGAGCAGGACTGGGCGG
CGGCCAAAGCGGTCGGACAGTGCTCCGAGAACGGGTGCGCATAGAAATTGCATCAACGCATATAGCGCTAGCAGCAC
GCCATAGTGACTGGCGATGCTGTCGGAATGGACGATATCCCGCAAGAGGCCCGGCAGTACCGGCATAACCAAGCCTA
TGCCTACAGCATCCAGGGTGACGGTGCCGAGGATGACGATGAGCGCATTGTTAGATTTCATACACGGTGCCTGACTG
CGTTAGCAATTTAACTGTGATAAACTACCGCATTAAAGCTTATCGATGATAAGCTGTCAAACATGAGAA

SUPPLEMENTARY INFORMATION REFERENCES

1. Mazzoni,E.O., Mahony,S., Closser,M., Morrison,C.A., Nedelec,S., Williams,D.J., An,D., Gifford,D.K. and Wichterle,H. (2013) Synergistic binding of transcription factors to cell-specific enhancers programs motor neuron identity. *Nat. Neurosci.*, **16**, 1219–1227.
